# Unraveling the molecular mechanism of photosynthetic toxicity of highly fluorescent silver nanoclusters to *Scenedesmus obliquus*

**DOI:** 10.1038/s41598-017-16634-5

**Published:** 2017-11-27

**Authors:** Li Zhang, Nirmal Goswami, Jianping Xie, Bo Zhang, Yiliang He

**Affiliations:** 1 School of Environmental Science and Engineering, Shanghai Jiao Tong University, No. 800 Dongchuan Road, Minhang District, Shanghai, 200240 China; 2 Department of Chemical and Biomolecular Engineering, National University of Singapore, 4 Engineering Drive 4, #03-18, Singapore, 117585 Singapore

## Abstract

While the discovery of numerous attractive properties of silver at the nanoscale has increased their demand in many sectors including medicine, optics, sensing, painting and cosmetics, it has also raised wide public concerns about their effect on living organisms in aquatic environment. Despite the continuous effort to understand the various aspects of the toxicity of silver nanomaterials, the molecular level understanding on their cytotoxicity mechanism to biological organisms has remained unclear. Herein, we demonstrated the underlying mechanism of the photosynthetic toxicity against green algae namely, *Scenedesmus obliquus* by using an emerging silver nanomaterial, called silver nanoclusters (defined as r-Ag NCs). By exploiting the unique fluorescence properties of r-Ag NCs along with various other analytical/biological tools, we proposed that the photosynthetic toxicity of r-Ag NCs was largely attributed to the “joint-toxicity” effect of particulate form of r-Ag NCs and its released Ag^+^, which resulted in the disruption of the electron transport chain of light reaction and affected the content of key enzymes (RuBP carboxylase/ oxygenase) of Calvin cycle of algae cells. We believe that the present study can also be applied to the assessment of the ecological risk derived from other metal nanoparticles.

## Introduction

The development of various synthetic methods to produce large quantities of nanomaterials, the detailed knowledge of their structures as well as the abundance of new physical and chemical properties at the nanoscale regime drive researchers to produce new and better nanomaterial-based products which can offer enormous benefits to the society^1–3^. As a consequence, many of these nanomaterials are now part of our daily life as they present in the form of cosmetics^4,5^, food packaging^6,7^, drug delivery systems^8–10^, therapeutics^11–14^, biosensors^15–18^, and so on^19–21^. Despite obvious benefits from the power of small sizes, the widespread applications of these materials in every sector of the society have raised significant concerns regarding their environmental impacts^22–24^.

A key example is silver nanoparticles (Ag NPs), which are now being produced in large scale with different sizes, shapes and functionalities in various sectors including biomedical science, daily life, wastewater treatment and industry^5,25–27^. Such widespread applications of these Ag NPs will inevitably cause their release into the environment and therefore, the study on the environmental fate and behavior of Ag NPs has become a broad subject of interest in recent times^28,29^. In fact, the majority of Ag NPs from commercial products are demonstrated to affect the aquatic ecosystems, in particular, to bacteria^30,31^, algae^32^, plant^33^, metazoan^34^, vertebrates or other aquatic organisms^35–37^. Therefore, studies governing the toxicity of Ag NPs to many of these aquatic organisms have been performed extensively and in some cases, the mechanisms of toxicity are well understood. Compare to other aquatic organisms, quite a few studies have investigated the effects of Ag NPs on algal growth and photosynthesis^38^. Algae, constituting the primary producer of the aquatic ecosystem, perform almost half of the photosynthesis on the earth and also play a very important role in the global carbon cycle^39,40^. Thus, assessing the potential harm of Ag NPs in algal growth and photosynthesis is of paramount interest.

To address this challenging issue, it is equally important to use a set of Ag NPs with high monodispersity, as it would constitute the toxicological results more relevant for understanding the current risks. However, the relatively polydisperse nature of many of the Ag NPs synthesized to date remains a major obstacle to their use as an ideal system for studying the toxicity effects of Ag NPs at molecular level^28,29^. Such a challenge can be addressed by using an emerging class of Ag NPs, generally called Ag nanoclusters (NCs). They are generally consisting of several to a hundred silver atoms with particle sizes less than 2 nm^41–43^. These ultrafine particles can be synthesized with complete monodispersity and thus, often represented by well-defined size and composition. In contrast to their large counterparts i.e., Ag NPs, they display different physiochemical properties such as HOMO–LUMO transition, quantized charging, strong photoluminescence etc^42,44–48^. All these unique properties of these Ag NCs together with their ultrafine size and structures made them an emerging functional material for many practical applications^49–54^.

To obtain better understanding of the toxicity of nanomaterials to aquatic organisms, it is also equally important to select the appropriate research methods. Currently, the majority of environmental toxicology of Ag NPs were performed by means of acute toxicity experiments (short-term exposure to high dosages) *in vivo* or cellular levels^55–57^. Ag NPs may have complex and dynamic process in aquatic environment including aggregation, oxidation, and reactions of silver with chloride ion and sulfur^28,58^. Therefore, chronic toxicity experiments (long-term exposure to low dosages) were ideally suited for the toxicological studies of Ag NPs to aquatic organisms. However, this type of researches are usually expensive and time-consuming, and also difficult to conduct in laboratory. Recently, molecular technology (e.g. transcriptome or metabolomics) have emerged as a useful tool to characterize the toxicity of nanoparticles to biological organisms^59–61^. Among which, RNA sequencing, regarded as the high-throughput sequencing technologies, was able to identify the specific transcriptomic responses of the aquatic organisms to Ag NPs or its degraded Ag ions^62–65^. This approach will help to not only investigate subtle differences in RNA level but also clarify the molecular mechanisms of the toxicity of Ag NPs to aquatic organisms.

In the present study, we have chosen a red emitting Ag NCs (defined as r-Ag NCs) as a model system to unravel the molecular mechanisms of photosynthetic toxicity against green algae namely, *Scenedesmus obliquus*. By making the most of advanced transcriptome technology as well as the combination of confocal laser scanning microscope (CLSM) basing on the strong fluorescence of the r-Ag NCs, we have demonstrated that r-Ag NCs toxicity mainly originated from particle-specific r-Ag NCs and its dissolved silver (I). This work will provide additional insights into the cytotoxicity mechanisms mediated by metal-based nanomaterials.

## Results and Discussion

### Synthesis, characterization and stability of r-Ag NCs

**Figure 1:**
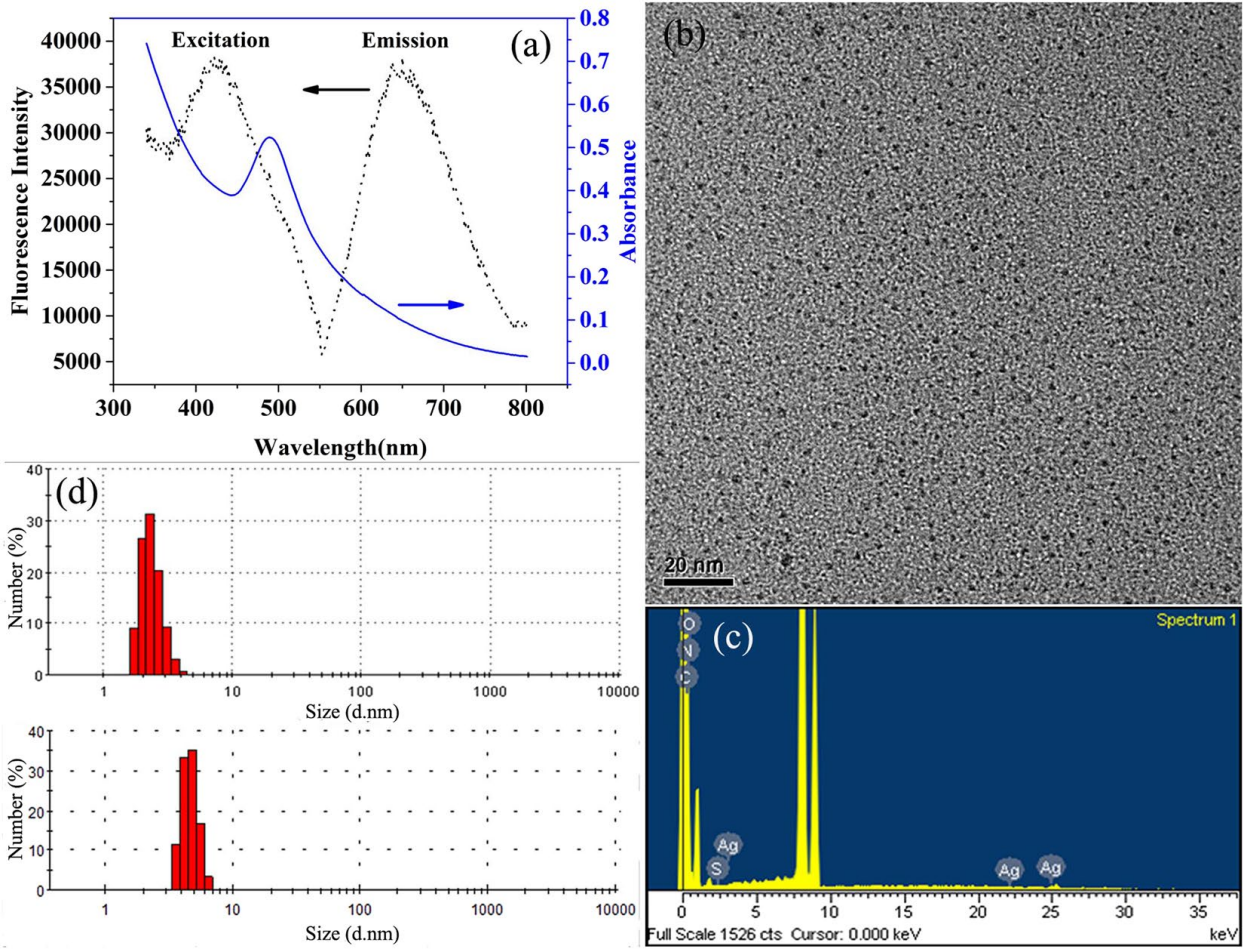
(**a**) UV-Vis absorption (blue line) and fluorescence (black line, λ_*ex*_ = 430 nm) spectrum of the as-synthesized r-Ag NCs. (**b**) TEM image of r-Ag NCs. The scale bar is 20 nm. (**c**) Energy-dispersive spectroscopy spectrum of r-Ag NCs. (**d**) Hydrodynamic diameter of r-Ag NCs dispersed in Milli-Q water (top panel) and SE culture medium (bottom panel).

Since our goal was to find out the Ag nanoparticle induced molecular mechanism of photosynthetic toxicity against green algae namely, *Scenedesmus obliquus*, we first prepared the well-defined r-Ag NCs solution following previous protocol^66,67^. The as-synthesized NC solution was light brown in color (Supplementary Fig. S2a), which showed a prominent absorbance peak at around 488 nm (Fig. 1a, blue line) in the UV-Vis spectrum. The strong red fluorescence was evident from the Supplementary Figure S2b. Nevertheless, the emission peak around 650 nm (Fig. 1a, black dot line) along with maximum excitation of 430 nm (Fig. 1a, black dot line) (in view of the presence of various well defined Ag NCs in solution), further corroborates that our as-synthesized material is indeed Ag NCs as their optical properties are in line with the previous report^66,67^. Such intriguing optical properties, especially fluorescence, are not observed in the case of well-commercialized Ag nanoparticles (>2 nm). We further verified their size through transmission electron microscopic (TEM) studies which revealed that the as-synthesized r-Ag NCs was nearly spherical in shape with average size less than 2 nm (Fig. 1b), and the synthesis of r-Ag NCs in our study was also confirmed by energy-dispersive spectroscopy spectrum (Fig. 1c). Similar average size was also observed in our dynamic light scattering (DLS) study, where pure r-Ag NCs in Milli-Q water showed hydrodynamic diameter ~3.43 ± 0.15 nm. The relatively bigger size observed in DLS was due to the presence of thiolate ligands and the water molecules on the NC’s surface which are not observed in TEM. To further check the stability of the as-synthesized NCs in SE culture medium, we performed the DLS study to verify their size and surface charge. The average hydrodynamic diameter of the as-synthesized NCs in SE culture medium was about 4.85 ± 0.21 nm, which was larger than that in Milli-Q water (Fig. 1d) due to the fact that the presence of electrolytes in the SE medium (Table S1) could induce the aggregation of NCs. The average zeta potential analysis revealed that r-Ag NCs were negatively charged in both pure water (−15.5 ± 0.36 mV) and SE medium (−34.5 ± 5.7 mV), indicating the r-Ag NCs were quite stable in SE culture medium and Milli-Q water.

After successful synthesis of r-Ag NCs, we have further assessed their stability in SE culture medium (Supplementary Fig. S3). In particular, we have evaluated the concentration of Ag^+^ released in the SE culture medium with time and based on these results, we have further designed the experimental schemes (see the experimental section for details).

### Effect of r-Ag NCs and dissolved Ag^+^ on the photosynthetic toxicity of algae

**Figure 2:**
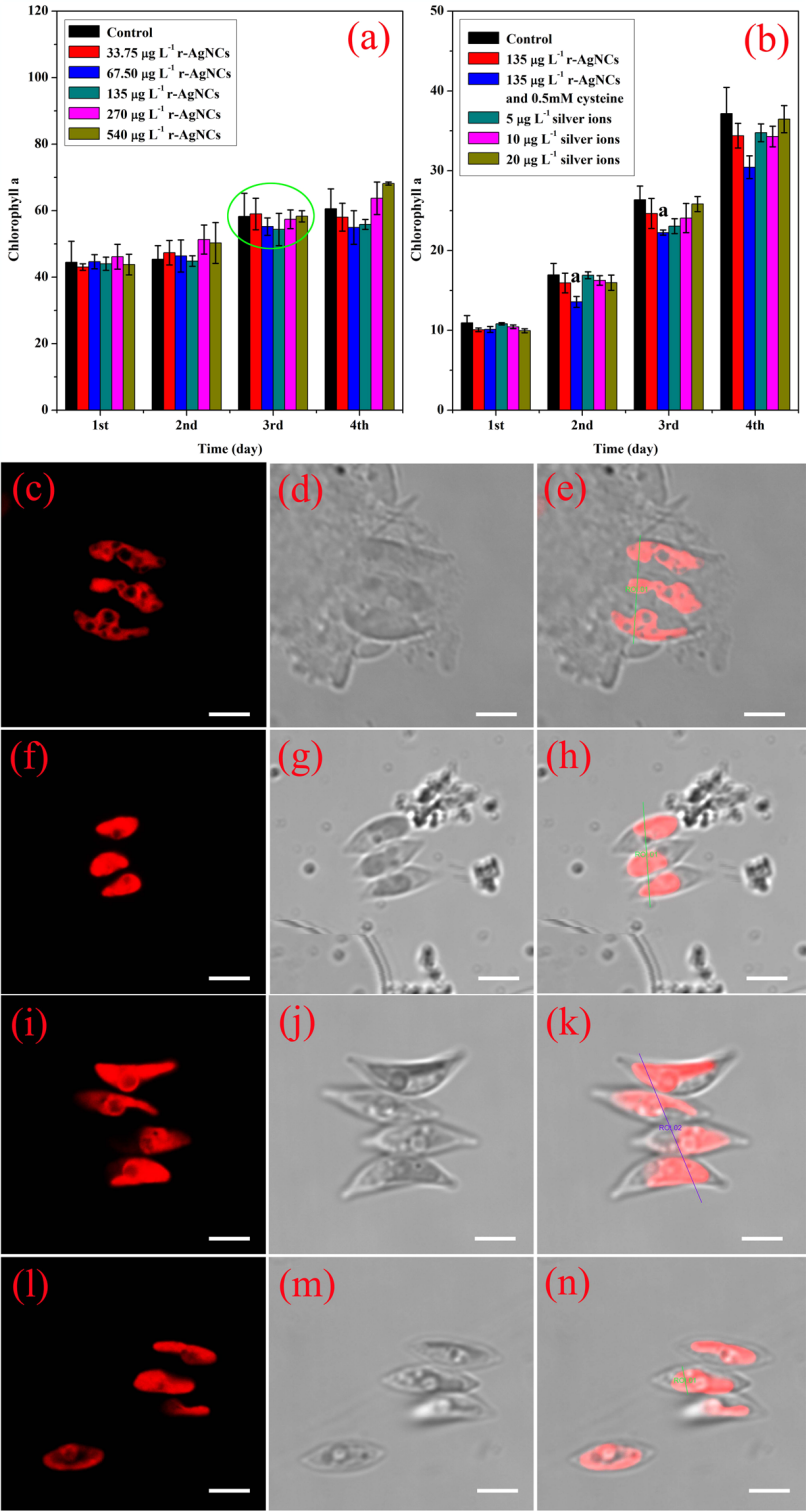
The chlorophyll α of *Scenedesmus obliquus* exposed to various concentrations of r-Ag NCs (**a**) and Ag^+^ (**b**) for 96 h. The controls were performed with SE culture medium containing no silver. Data were shown as mean value ± standard deviation for technical triplicates. Statistical analyses were carried out using one-way ANOVA followed by independent *t*-test (^a^ *P* < 0.05). Representative confocal laser scanning microscope images (**c**–**n**) of *Scenedesmus obliquus* in response to exposure to various concentration of r-Ag NCs and Ag^+^ for 96 h. Each row (from top to bottom) represented algae cells exposed to controls, r-Ag NCs, r-Ag NCs containing L-cysteine and AgNO_3_, respectively. Each column (from left to right) represented confocal fluorescence images (red pseudocolour) at 458 nm excitation, bright field images and merged images, respectively. The straight lines of images (**e**,**h**,**k** and **n**) showed the fluorescence intensity of designated spots of algae cells. Scale bar is 2.5 μm.

The photosynthetic activities of *Scenedesmus obliquus* exposed to various concentration of r-Ag NCs and Ag^+^ have been adversely affected. Compared to the controls, where only Ag^+^ of approximately equivalent concentration were used, the chlorophyll α content did not show any obvious dose–response up to 48 h (Fig. 2a) in case of r-Ag NCs. However, with the increase in the concentration of r-Ag NCs, a clear U type dose-dependent manner from 48 to 96 h was observed, where 135 μg L^−1^ r-Ag NCs displayed the maximum inhibition of chlorophyll α at 72 h. Notably, the addition of 0.5 mM L-cysteine to the r-Ag NCs solution resulted in relatively lower chlorophyll α content than that of algae cells exposed to r-Ag NCs without L-cysteine (Fig. 2b). This phenomenon can be explained by the strong chelating affinity between Ag^+^ and thiol groups of functional proteins of algal cell membranes, which cause disturbance of absorbance and transportation of r-Ag NCs^28,68^. Moreover, with the increasing concentration of Ag^**+**^ (from 5.0 to 20 μg L^−1^), the content of chlorophyll α was gradually decreased within 24 h of exposure and followed an obvious dose-response up to the time point of 48 h. However, after this time chlorophyll α content returned to control levels, indicating that the algae cells have mounted a detoxifying response and contribute to the recovery of the photosynthetic activities of algae cells.

Several other photosynthetic parameters have also been taken into account in our study (Supplementary Fig. S4). In fact, when exposed to various concentrations of r-Ag NCs and Ag^+^, some photosynthetic parameters showed a clear dose-dependent manner and the inhibition of Ag^**+**^ to cell membranes like the chlorophyll α content (e.g. Supplementary Fig. S4c,d). Sometimes, both r-Ag NCs and Ag^+^ even increased the photosynthetic parameters of algae cells (e.g. Supplementary Fig. S4f–h). These data suggested that r-Ag NCs and Ag^+^ had complicated effects on the photosynthetic activities of algae cells due to the compound body of chlorophyll α and proteins of photosystem (PSII and PSI). Therefore, to further substantiate the detailed molecular mechanisms that affect the photosynthetic activities, we have also analyzed the transcriptome of *Scenedesmus obliquus* treated with r-Ag NCs and Ag^+^ (see below in the text).

Since it is equally important to verify whether the r-Ag NCs indeed internalized by the algae cells or not, confocal microscopic studies were conducted. The confocal fluorescent images were taken by using a 458-nm laser under low voltage conditions. In striking contrast to the auto-fluorescence of chlorophyll α of the control experiments (Fig. 2c,e; Supplementary Fig. S5a), fluorescence intensity (Fig. 2h,k; Supplementary Fig. S5b,c) of the algae cells exposed to 135 μg L^−1^ r-Ag NCs solution (with or without L-cysteine) were stronger. On the other hand, the fluorescent intensity of algae cells treated with Ag^+^ was decreased slightly when compared with the control (Fig. 2l,n; Supplementary Fig. S5d). These data suggested that r-Ag NCs were indeed internalized by the algal cells and dispersed in the entire cytoplasm. The internalization of r-Ag NCs in the entire cytoplasm was further verified by z-axis scanning images of algae cells (Fig. S6). When the z-axis moved from the top to the bottom of the sample, the confocal cross sectional images of the cells become larger and brighter gradually, then smaller and darker, and finally nearly invisible, indicating that r-Ag NCs have been internalized into the cytoplasm rather than adsorbed on the algal cells surface. The internalization of r-Ag NCs observed from confocal images can also be evidenced by the TEM images (Fig. S7).

### Transcriptome data analysis

The concentration and integrity of RNA were first determined by UV-Vis spectrophotometer and agarose gel electrophoresis (Supplementary Table S2 and Fig. S8). To obtain the various silver-treated *Scenedesmus obliquus* transcriptome expression profile, four cDNA libraries were constructed: (i) the controls (Sample A), (ii) algae cells exposed to concentration of 135 μg L^−1^ r-Ag NCs (Sample B), (iii) algae cells exposed to concentration of 135 μg L^−1^ r-Ag NCs containing 0.5 mM of L-cysteine (Sample C) and (iv) algae cells exposed to concentration of 10 μg L^−1^ silver (Sample D). The paired-end raw reads and clean reads for four samples were available in supporting information (Supplementary Table S3). Transcript *De novo* assembly was conducted using the clean reads by Trinity software and the summary of all contigs, transcripts and unigenes was listed in Supplementary Table S3. Overall, 26,570 unigenes were obtained for the further analysis of differentially expressed genes. The annotation of unigenes were summarized in Supplementary Figure S9~S12, and approximately 1800,1500 and 300 unigenes were annotated into the term of energy process of GO, eggNOG and KEGG database (blue arrow), respectively.

**Figure 3:**
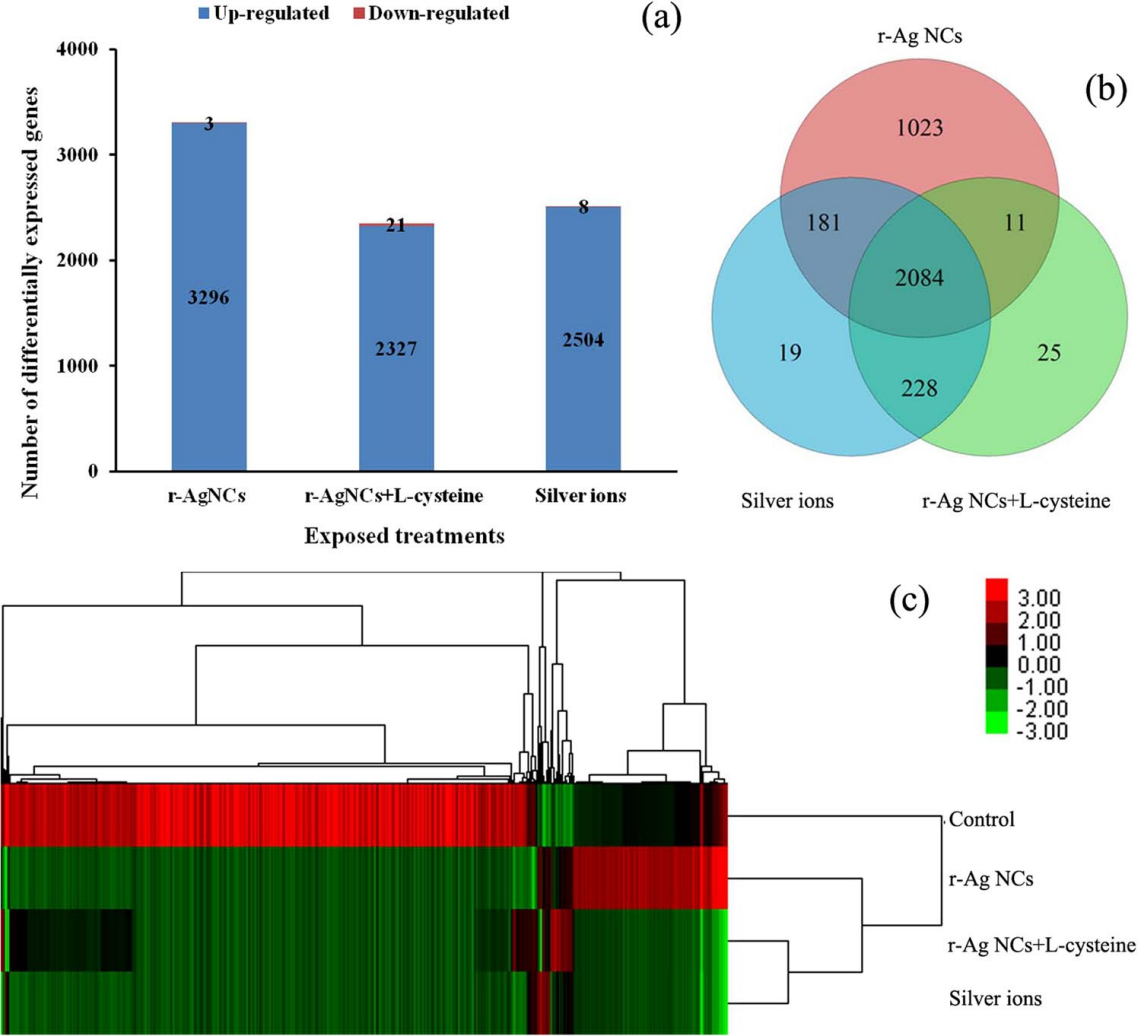
Differentially expressed genes in the silver-treated *Scenedesmus obliquus*. (**a**) The number of differentially expressed genes (≥2-fold up/down-regulated, P < 0.05) derived from *Scenedesmus obliquus* after exposure for 96 h to r-Ag NCs,r-Ag NCs + L-cysteine and Ag^+^ as compared to control. The numbers of up- and down-regulated genes for each of the silver treatments are indicated on the bars. (**b**) Venn diagram showing numbers of differentially expressed genes (≥2-fold up/down-regulated, P < 0.05) from *Scenedesmus obliquus* after exposure to r-Ag NCs, r-Ag NCs + L-cysteine and Ag^+^. (**c**) Heatmap of differentially expressed genes (≥2-fold up/down-regulated, P < 0.05) from *Scenedesmus obliquus* exposed to control, r-Ag NCs, r-Ag NCs + L-cysteine and Ag^+^. The heatmap was established using all differentially expressed genes by Cluster 3.0/TreeView software. The level of gene expression was shown using a color gradient from green (low expression) to red (high expression).

Statistical analysis of the differentially expressed genes was performed between each of the silver treatment and the control one. The “MA plot” pictures of differentially expressed genes between each silver treatment and the controls were shown in Supplementary Figure S13. It was evident that the levels of expression of most of the differentially expressed genes from algal cells exposed to each silver treatment were down-regulated when compared with the control one, and further illustrated that both r-Ag NCs and its released Ag^+^ had toxic effects to algal cells at the transcriptional level. The number of the differentially expressed genes of each silver treatment were almost consistent with the overlap between the differentially expressed genes for each treatment (Fig. 3a,b). The heatmap of differentially expressed genes between each of the silver treatment library was presented in Fig. 3c which revealed that there were significant differences in gene expression levels for the differentially expressed genes between the various silver treatment and the control as well as there were some consistency in gene expression between particles-specific r-Ag NCs and its dissolved Ag^+^ according to the condition tree of the heatmap.

As a primary producer under aquatic environment, energy metabolism is one of the most important life characteristics of algal cells. Therefore, we investigated the significant changes of energy metabolism in gene expression after exposure of *Scenedesmus obliquus* to silver for 96 h. Some of the differentially expressed genes were detected to be relevant to each silver treatment compared to the control one (135 μg L^−1^ r-Ag NCs − 67 genes; 135 μg L^−1^ r-Ag NCs containing 0.5 mM of L-cysteine − 62 gene; Ag^+^ − 65 genes), largely reflecting gene expression responses to the energy metabolism of *Scenedesmus obliquus*. Expression of these genes encoding functional molecules of cellular components were closely associated with the energy transformation process of algae cells such as, photosynthesis, oxidative phosphorylation and nitrogen metabolism and so on, indicating the processes of energy metabolism of *Scenedesmus obliquus* were regulated on the molecular levels after exposed for 96 h to r-Ag NCs and its dissolved Ag^+^.

### Common features in the effects of exposure to r-Ag NCs and Ag^+^ on algae gene expression

**Figure 4:**
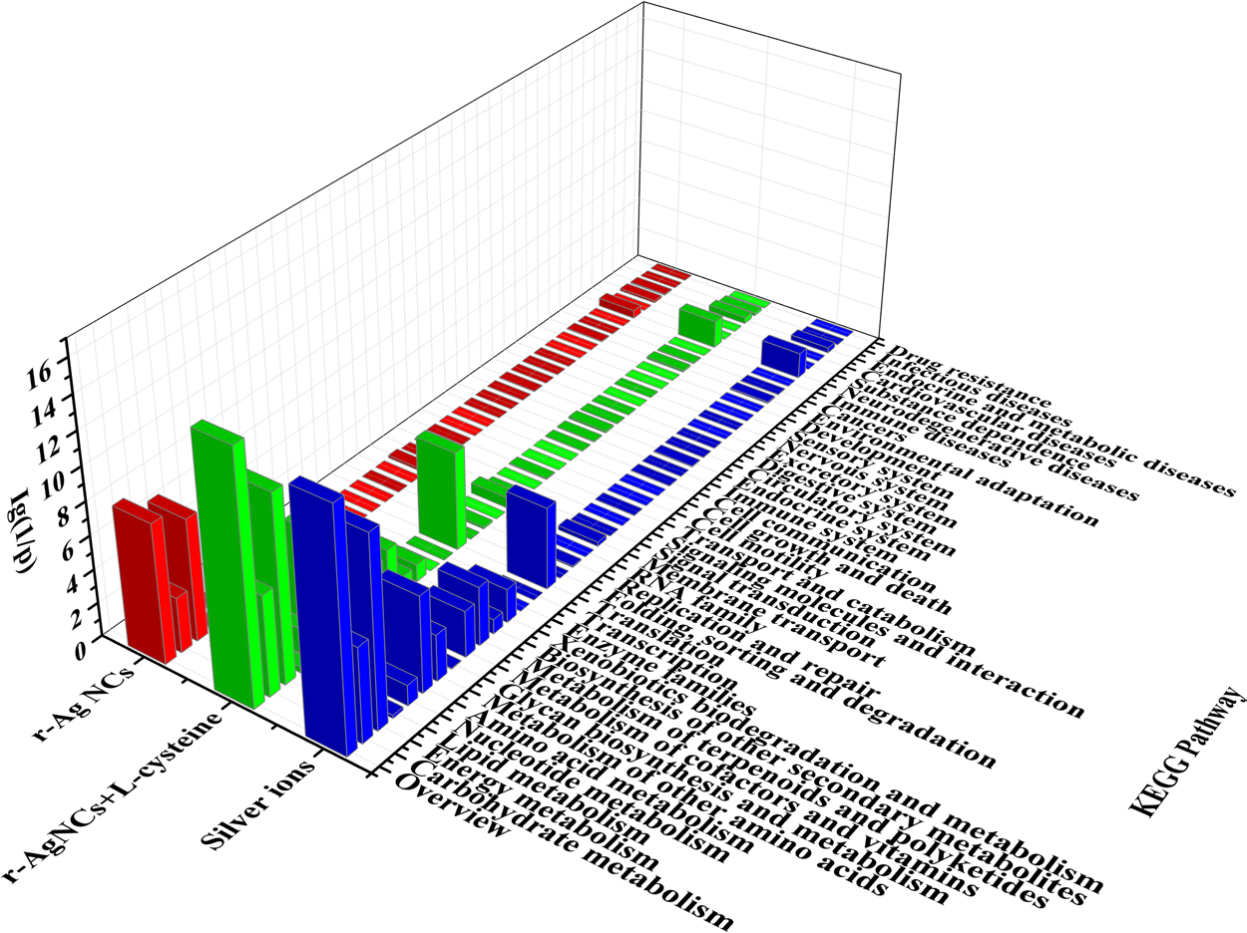
KEGG pathway enrichment analysis of differentially expressed genes after exposure of *Scenedesmus obliquus* to silver for 96 h. The enrichment analysis of differentially expressed genes was performed between each of silver treatment and the controls (adjusted *P* values < 0.05) using all the unigenes as a background.

The changes in gene expression in *Scenedesmus obliquus* exposed for 96 h to r-Ag NCs (with and without L-cysteine) and silver ions (control) have been explored by determining the enrichment in GO terms (in terms of GOSlim in our study, Supplementary Fig. S14) and KEGG pathway (Fig. 4). Enrichment analysis of differentially expressed genes was performed to obtain a global overview of the biological effects of each type of silver treatments. The most significantly representative GO terms (metabolic process) and KEGG pathways (energy metabolism) remained common across all three silver treatments, and a very significant overlap was observed between differentially expressed genes in response to the three forms silver considered in our study. On the basis of this overlap, we anticipate that the toxicity of r-Ag NCs to algae cells was predominantly attributed to the association between the nanoscale and the ionic form silver.

In a previous study, we demonstrated similar results that both the nanoscale size of particles and their decomposition products contribute to the cellular toxicity mechanism upon the exposure of *Scenedesmus obliquus* to r-Ag NCs^69^. Similar observations were also evident from other studies. For example, the case of the Copper Transport Protein 2(CTR2), where mRNA levels of the green alga *C*. *reinhardtii* were strongly stimulated by both a small silver nanoparticles (size~5 nm, with a polyacrylate coating) and the Ag^+ ^^62^. We therefore anticipate that there is a combination of r-Ag NCs particles and Ag^+^ inside the algae cells (see Fig. 2 and Supplementary Fig. S7). For many of the differentially expressed genes derived from *Scenedesmus obliquus* after exposure to r-Ag NCs (without L-cysteine), the changes in gene expression (in terms of fold change) were almost consistent with those exposed to r-Ag NCs (containing L-cysteine) and Ag^+^, further confirmed that r-Ag NCs and its dissolved Ag^+^ could jointly mediate the cytotoxicity. Moreover, the GO and KEGG pathway enrichment analysis were common to all silver treatments (Supplementary Fig. S14; Fig. 4). These changes are also consistent with previous report such as the transcriptional response of the model plant, *Arabidopsis thaliana*, exposed to Ag NPs and Ag^+^ using whole genome cDNA microarrays^70^.

Energy metabolism of *Scenedesmus obliquus*, including photosynthesis and oxidative phosphorylation, is a complex process and both these process were affected consistently after exposure to all kinds of silver treatments. As shown in Fig. 4, the enrichment of the differentially expressed genes in KEGG pathway (in term of energy metabolism) was more significant than other metabolic pathways (adjusted *p* < 0.05). Moreover, Ig(1/*p*) of the term of metabolic process was larger than that of the mitochondrion (Supplementary Fig. S14, adjusted *P* < 0.05). In other words, the enrichment of the differentially expressed genes in GOSlim (in term of metabolic process) was more pronounced than that of the mitochondrion, indicating both r-Ag NCs and its dissolved Ag^+^ had more influences on the photosynthesis of the algal cells rather than the oxidative phosphorylation.

### Photosynthesis metabolic pathways

**Figure 5:**
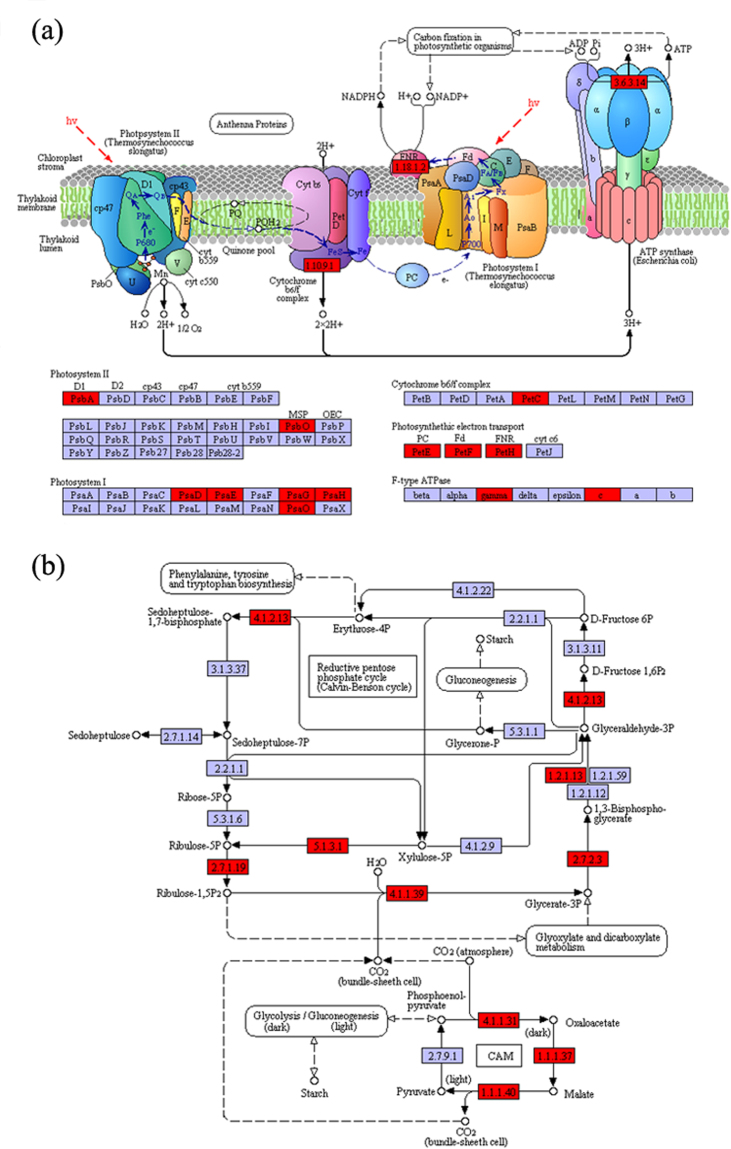
Effects of each silver treatment on differentially expressed genes associated with photosynthesis of *Scenedesmus obliquus*. Shades of red, light blue and white indicate up-regulation of target genes of controls, no differential expression of genes (Unigenes) and gene products not belong to the photosynthesis of *Scenedesmus obliquus*, respectively. (**a**) Effects of exposure to r-Ag NCs without L- cysteine for 96 h on the light reaction of photosynthesis of *Scenedesmus obliquus*. (**b**) Effects of exposure to r-Ag NCs without L- cysteine for 96 h on the photosynthetic carbon reduction cycle (Calvin cycle) of *Scenedesmus obliquus* (Adapted from the carbon fixation pathway map in the KEGG database). The number in the boxes represents the identifier of enzyme involved in the pathway according to the principles of classification of Enzyme Commission. Illustrations of the light reaction and photosynthetic carbon reduction cycle of *Scenedesmus obliquus* exposed to r-Ag NCs with L- cysteine and Ag^+^ treatments are available in Table S4. The differentially expressed genes in photosynthetic pathways (light reaction and Calvin cycle) were determined by using the Kyoto Encyclopedia of Genes and Genomes (KEGG) database^74^.

Exposure to all kinds of silver treatments caused the photosynthesis of *Scenedesmus obliquus* to be adversely affected (Fig. 5). The light reaction of photosynthesis is a complicated metabolic pathway and is composed of a series of reaction center complexes of the photosynthetic electron transport chain, including photosynthesis II, I, cytochrome b6/f and ATP synthase, which plays a key part in the synthesis of ATP in aquatic organism. Following exposure for 24 h to all Ag treatments, the gene expression data demonstrated that many of the genes encoding for the complexes of light reaction of photosynthesis were down-regulated as compared to control (Fig. 5a and Supplementary Table S4). Among all these genes, the psbA was especially sensitive to r-Ag NCs and Ag^+^ exposure, which encoded for photosystem II P680 reaction center D1 protein containing primary electron donor Z (Tyr^−1^61), reaction center pigment (P680) and primary electron accepter (Pheophytin). The psbO encoded for photosystem II oxygen-evolving enhancer protein 1, which involved the released oxygen of photosynthesis and electron donor for Z. The petF and ATPF0C, encoded for Ferredoxin and subunit c of ATP-synthase, were also down regulated at the transcriptome level. These genes are particularly relevant because the electron transport chain is comprised of several reaction center complexes, encoded by a large number of the chloroplast and the nuclear genome, such as psbA, psbO and petF and so on. The down-regulated genes encoded for the reaction center complexes proteins which had not only influences on the utilization efficiency of light energy (Supplementary Fig. S4c,d) but also on the electrochemical proton gradient across the thylakoid membrane of chloroplast, and further affected the synthesis of ATP. This effect is in accord with previously study reporting a down-regulation of this pathway following Ag^+^ ions exposure in *C*. *reinhardtii* ^71^. Overall, a down-regulation of these key genes encoded for the reaction center complexes proteins suggest the light reaction of photosynthesis of *Scenedesmus obliquus* was influenced synchronously by the exposure to r-Ag NCs and its dissolved Ag^+^.

Apart from the damage on the light reaction of photosynthesis, exposure to r-Ag NCs and Ag^+^ also caused changes in the Calvin cycle and showed similarly significant alterations in the expression of genes (Fig. 5b and Supplementary Table S4). The Calvin cycle consists of three stages of carbon dioxide fixation, reduction and regeneration of ribulose – 1,5 – bisphosphate (RuBP) and features eleven-enzymatic reaction for the biosynthesis of carbohydrate. The key enzymes, such as RuBP carboxylase/oxygenase (Rubisco) containing eight small subunits encoded by gene rbcs (nuclear genes), were down regulated. This regulation indicates that the carbon fixation was probably also regulated and can be regarded as a significant regulatory site in Calvin cycle, resulting in declines in the 3-phosphoglyceric acid synthesis. Previous analysis mentioned above have associated the loss of the proton gradient arising from the disruption of the electron transport chain with the synthesis of ATP, which will be utilized as assimilatory power for the reduction of carbon dioxide in the Calvin cycle. Taken together, it can be stated that the disturbance of light reaction of photosynthesis by the exposure to all silver treatment have an influence on the Calvin cycle.

Previous studies have reported the impact of r-Ag NCs on various organisms^51,66,69,72^. However, it is still unclear whether r-Ag NC serves as a direct cause of toxicity and toxicity mechanisms or not. Yuan *et al*. investigated the underlying mechanism of the antimicrobial ability of r-Ag NCs after exposure of r-Ag NCs to *P*. *aeruginosa* for 14 h and suggested that r-Ag NCs could eliminate pathogenic bacteria by means of generating intracellular reactive oxygen species (ROS)^66^. However, in our recent study, where *Scenedesmus obliquus* was exposed to various concentrations of the r-Ag NCs and Ag^+^, we have excluded the possibility of ROS production^69^. The antimicrobial activity of r-Ag NCs on both gram negative (i.e., *P*. *aeruginosa*, and *E*. *coli*) and gram positive (i.e., *B*. *subtilis*, and *S*. *aureus*) bacteria could be originated from the dissociation of Ag^+^ ions, as reported by some other studies^51^. In consideration of similarly significant changes in the expression of differentially expressed genes in *Scenedesmus obliquus* after exposure to r-Ag NCs and Ag^+^ and observation results derived from confocal fluorescence images and TEM images in our study, it might be possible that r-Ag NCs entering the algae cells could gradually degrade into Ag^+^ which further mediated r-Ag NCs cytotoxicity. A similar conclusion was made for larger particles. The study revealed that the cytotoxicity of 20 nm silver nanoparticles (Ag NPs) on human monocyte (THP-1) is largely due to the chemical transformation of particulate silver from Ag NPs to Ag^+^ ions, indicating this could be a potential toxicity mechanism^73^.

### Transcriptome validation of photosynthesis specific genes

**Figure 6:**
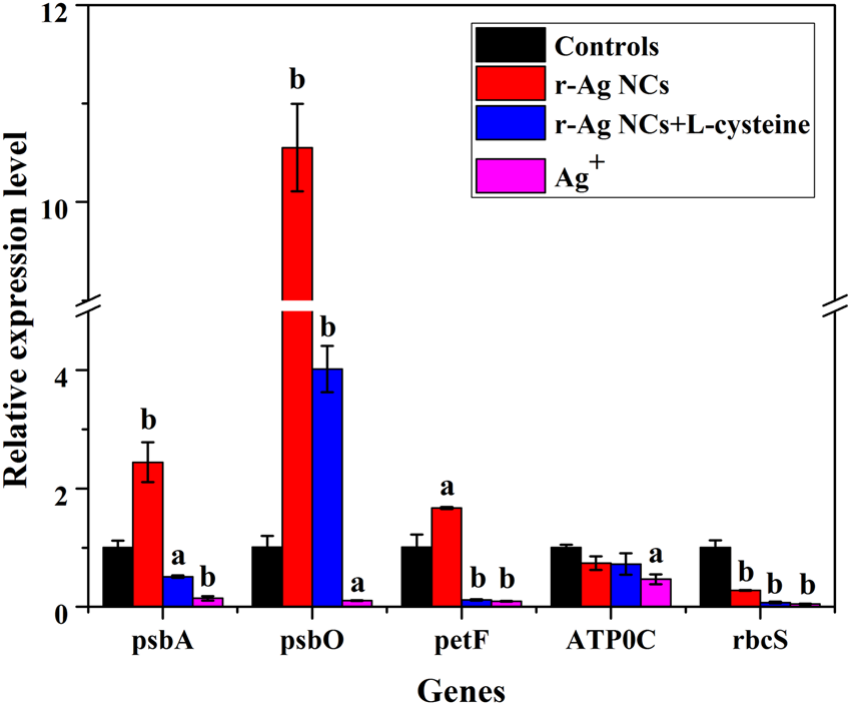
qRT-PCR analysis of expression levels for several representative genes in the light reaction and photosynthetic carbon reduction cycle of *Scenedesmus obliquus* exposed to controls and silver treatments. Substantial differences were determined by one-way ANOVA test followed by independent *t*-test. (^a^ *P* < 0.05; ^b^ *P* < 0.01).

Independent qRT-PCR experiment was performed to confirm the combined effects of r-Ag NCs and Ag^+^ to the photosynthesis of *Scenedesmus obliquus*. As shown in Fig. 6, the expression levels of genes (psbA, petF and ATPF0C) involved in the light reaction of both r-Ag NCs + L-cysteine as well as Ag^+^ exposed algae cells were less than that of the control ones. However, expression levels of genes mentioned above (r-Ag NCs without L-cysteine), especially for the gene psbO, were higher than that of the control one, suggesting that Ag NCs and their released Ag^+^ could stimulate the increase of expression levels of genes. Similarly, in the process of Calvin cycle, expression levels of genes (rsbS) from algal cells exposed to three kinds of silver treatment were also less than that of the control one. Taken together, qRT-PCR analysis further confirmed that both r-Ag NCs and its dissolved free Ag^+^ can cause immediate adverse effects on the photosynthesis of algal cells and thus validated the “joint-toxicity” effect mediated by r-Ag NCs and its dissolved Ag^+^.

## Conclusions

To summarize, we demonstrated the r-Ag NCs-mediated photosynthetic toxicity on *Scenedesmus obliquus* cells and the cytotoxic mechanism of r-Ag NCs at transcriptome level. The r-Ag NCs cytotoxicity is largely due to the “joint-toxicity” effect of particulate form of r-Ag NCs and its dissolved Ag^+^ on the photosynthesis of algae cells. The light reaction of photosynthesis was inhibited due to the disruption of the electron transport chain caused by a down-regulation of several key genes (e.g. psbA, petF and ATPF0C) encoded for the reaction center complexes proteins. Meanwhile, the down-regulation of gene rbcs regulates the expression of key enzymes, especially RuBP carboxylase/ oxygenase (Rubisco), and further lead to the damage on the Calvin cycle, which is also regulated by the reduction of ATP arising from inhibition of light reaction. Whether the r-Ag NCs entering the algal cells could gradually degrade into Ag^+^ and further mediate the cytotoxic effects of r-Ag NCs, it is worthwhile to investigate this potential toxicity mechanism in future research. The combined data from the current study would not only help us to understand r-Ag NCs-mediated cytotoxicity, but also shed light on our understanding on the underlying toxic mechanisms and nanosafety assessments of metal nanoparticles.

## Materials and Methods

### Materials

Milli-Q water (18.2 MΩ.cm/25 °C) was used throughout this study. All glassware were washed with 0.05 M nitric acid, rinsed with Milli-Q water, and dried in an oven overnight. AgNO_3_, (99.9999%), L-glutathione reduced (GSH) and sodium borohydride (NaBH_4_) were purchased from Sigma-Aldrich. L-cysteine (≥ 99%) was purchased from Beijing Solarbio Science & Technology Co., Ltd. The other reagents (analytical reagent) were purchased from China National Pharmaceutical Group Corporation.

### Algae culture

Green algae namely, *Scenedesmus obliquus* (No. FACHB-417) purchased from the Institute of Hydrobiology (Wuhan City, China), were cultured in Bristol’s solution (SE culture medium, Supplementary Table S1). Prior to each algae culture, all glassware and SE culture medium were sterilized in autoclave instrument (121 °C, 30 min).

### Synthesis and characterization of r-Ag NCs

The synthesis of fluorescent r-Ag NCs was performed following the previous procedures with slight modification (See Supplementary experimental methods)^66,67^. The r-Ag NCs solutions were stored at 4 °C and used for further experiments. Transmission electron microscopy (TEM) images of the r-Ag NCs were taken with a JEOL JEM-2010. Hydrodynamic diameter and Zeta potential of r-Ag NCs in Milli-Q water and SE culture medium were determined by a Zetasizer (Malvern Nano series, UK). The fluorescent spectrum was measured by the QuantaMaster™ fluorescence spectrofluorometer (Photon Technology International, USA). Further details on the dissolution of r-Ag NCs in SE culture medium and Milli-Q water can be found in the Supplementary experimental methods and Fig. S2.

### Photosynthetic toxicity of r-Ag NCs and its released Ag^+^ to algae

The following experimental programs were conducted to investigate the photosynthetic toxicity of r-Ag NCs and its released Ag^+^ to *Scenedesmus obliquus*. (1): algae (5~10 × 10^5^ cells mL^−1^) were exposed to r-Ag NCs concentrations of 0.00, 33.75, 67.5, 135, 270 and 540 μg L^−1^, respectively (silver atom based). (2): L-cysteine, which is known to chelate Ag^+^, was used to inhibit the released Ag^+^ and further distinguish the effects of r-Ag NCs and its released Ag^+^. Algae (5~10 × 10^5^ cells mL^−1^) were then treated with the experimental solutions of r-Ag NCs (0.00, 135, and 135 μg L^−1^ containing 0.5 mM of L-cysteine) and Ag^+^ (AgNO_3_, 5.0, 10.0 and 20.0 μg L^−1^). Exposure concentration (135 μg L^−1^) was taken fully into account in our study so that exposure was as environmentally relevant as possible according to previous study (Ag NPs, 0.1~146 μg L^−1^)^29^. The concentration range of Ag^+^ released from r-Ag NCs (μg L^−1^) was approximately equal to that of Ag^+^ (5.0, 10.0 and 20.0 μg L^−1^) dissolved from AgNO_3_ (Supplementary Fig. S3). The controls were carried out with SE culture medium containing no silver. Each treatment was performed in biological triplicate.

After exposure, the photosynthetic activity of algae cells, such as chlorophyll α, Fv/Fm (Photosynthetic Yield (PSII) = (F_m_ − F_0_)/F_m_, where F_m_ and F_0_ are the maximum fluorescence and the normal fluorescence, respectively), α (Utilization efficiency of light energy), rETR_Max_ (Maximum relative electron transport rate) and I_k_ (Saturated light intensity), were recorded on a PHYTO-PAM Phytoplankton Analyzer (WALZ, Germany) at 24, 48, 72 and 96 h, respectively. Transmission electron microscopy (TEM) (See Supplementary experimental methods) and confocal laser scanning microscope (CLSM) were also utilized to observe the internalization of r-Ag NCs.

### RNA sequencing

Total RNA was extracted from algae cells using the trizol reagent according to the manufacturer’s instructions (Invitrogen, USA). The concentration and integrity of RNA were determined by a Nanodrop 2000 Spectrophotometer (Nanodrop, USA) and agarose gel electrophoresis, respectively. After the cDNA libraries were constructed with PCR amplification, the libraries were then sequenced with Illumina Nextseq. 500 platform. Further details are available in the Supplementary experimental methods.

### Transcriptome assembly processing and annotation

Raw sequencing reads were cleaned to obtain high quality reads by removing adapter sequences and low-quality sequences (reads with a base quality less than 20) from the 3′and 5′ends of the remaining reads. Quality reads were *de novo* assembled into contigs and butterfly to form the final transcripts using Trinity software (http://trinityrnaseq.sf.net). Subsequently, the transcripts were subjected to BLAST research (http://www.ncbi.nlm.nih.gov/) against NCBI non-redundant protein database (http://www.ncbi.nlm.nih.gov/) with an E-value threshold of 1e^−5^, and the top-hit transcripts were selected as unigenes. Functional annotations were performed by comparing the assembled unigenes with public databases. We searched all the unigenes against NCBI non-redundant protein database, Gene Ontology database (GO, http://geneontology.org/), Evolutionary genealogy of genes: Non-supervised Orthologous Groups (eggNOG) database (http://eggnog.embl.de/) and Kyoto Encyclopedia of Genes and Genomes (KEGG) database (http://www.genome.jp/kegg/) using NCBI Blast software. The detailed data of the transcriptome assembly were given in the Supplementary Table S3.

### Analysis of the differentially expressed unigenes

To detect the differentially expressed unigenes between each of the r-Ag NCs treatments and the controls in our study, reads per kilobase of exon model per million mapped reads value (RPKM) were employed to normalize the gene expression levels. False discovery rate (FDR) < 0.05 was used as the threshold of *p*-value in multiple test to assess the significance difference of gene expression. Differentially expressed unigenes between the r-Ag NCs treatments and the controls were identified using DESeq software (http://www-huber.embl.de/users/anders/DESeq, |fold change| > 2 and *P*-value < 0.05). GO enrichment analysis were carried out for lists of differentially expressed unigenes between each r-Ag NCs treatment and the control using the Goatools software (https://github.com/tanghaibao/GOatools), GO terms for Biological Process, Cellular Component, and Molecular Function were considered significantly enrichment when *P*-value < 0.05 was observed. Enriched KEGG pathways of differentially expressed unigenes (adjusted *P* < 0.05) were determined using the KOBAS software (http://kobas.cbi.pku.edu.cn/home.do). KEGG Orthology (KO) metabolic pathways analysis was performed through the use of the KEGG database (http://www.genome.jp/kegg/tool/map_pathway2.html), based on differentially expressed unigenes (adjusted *P* < 0.05), which represent molecular interactions and reaction network of algae cells. Cluster analysis of differentially expressed unigenes was conducted by using Cluster3.0/TreeView software (http://bonsai.hgc.jp/~mdehoon/software/cluster/manual/index.html).

### Real-time reverse transcription PCR (qRT-PCR)

Validation of transcriptome data was conducted by means of an independent qRT-PCR experiment (See Supplementary experimental methods). Four target genes (psbA,psbO,petF and ATPF0C) significantly down-regulated in light reaction of photosynthesis and a target gene (rbcS) significantly down-regulated in photosynthetic carbon reduction cycle upon exposure to both r-AgNCs (without and with L-cysteine) and Ag^+^ were selected from differentially expressed genes. Gene’s expression levels were estimated using SYBR Green qPCR master mix (KAPA) on StepOne Plus qRT-PCR System (Applied Biosystems) (see Supplementary experimental methods). α-tubulin was used as a reference gene for relative qualification. All primer sequences for qRT-PCR analysis are available in the Supplementary Table S5.

### Other statistical analysis

Statistical analyses for photosynthetic toxicity of algae were performed using the origin 8.0 software. The two-tailed Student’s *t* test or one-way ANOVA test was used to analyze the significance of mean difference among groups compared to the control. Data were shown as the mean value ± standard deviation (n = 3), and *P* value of less than 0.05 was considered as statistical significant.

## Electronic supplementary material

Supplementary Information

## References

[1] Roco MC, Mirkin CA, Hersam MC (2011). Nanotechnology research directions for societal needs in 2020: summary of international study. J. Nanopart. Res..

[2] Yetisen AK (2016). Nanotechnology in Textiles. ACS Nano.

[3] Kagan CR (2016). Nano Day: celebrating the next decade of nanoscience and nanotechnology. ACS Nano.

[4] Gondikas AP (2014). Release of TiO_2_ nanoparticles from sunscreens into surface waters: a one-year survey at the old Danube recreational Lake. Environ. Sci. Technol..

[5] Chernousova S, Epple M (2013). Silver as antibacterial agent: ion, nanoparticle, and metal. Angew. Chem. Int. Ed. Engl..

[6] Kumar SM (2016). P. Controlled biodegradation of polymers using nanoparticles and its application. RSC Adv..

[7] Díez-Pascual AM, Díez-Vicente AL (2015). Antimicrobial and sustainable food packaging based on poly(butylene adipate-co-terephthalate) and electrospun chitosan nanofibers. RSC Adv..

[8] Xue X (2013). Nanoscale drug delivery platforms overcome platinum-based resistance in cancer cells due to abnormal membrane protein trafficking. ACS Nano.

[9] Wang X (2013). Glutathione-triggered “off-on” release of anticancer drugs from dendrimer-encapsulated gold nanoparticles. J. Am. Chem. Soc..

[10] de Faria PC (2014). Oxidized multiwalled carbon nanotubes as antigen delivery system to promote superior CD8^+^ T cell response and protection against cancer. Nano Lett..

[11] Lee N (2015). Iron oxide based nanoparticles for multimodal imaging and magnetoresponsive therapy. Chem. Rev..

[12] Misra SK, Ohoka A, Kolmodin NJ, Pan D (2015). Next generation carbon nanoparticles for efficient gene therapy. Mol. Pharm..

[13] Mieszawska AJ, Mulder WJ, Fayad ZA, Cormode DP (2013). Multifunctional gold nanoparticles for diagnosis and therapy of disease. Mol. Pharm..

[14] Lucky SS (2015). Titania coated upconversion nanoparticles for near-infrared light triggered photodynamic therapy. ACS Nano.

[15] Bai J, Zhou B (2014). Titanium dioxide nanomaterials for sensor applications. Chem. Rev..

[16] Ma W, Xu L, Wang L, Kuang H, Xu C (2016). Orientational nanoparticle assemblies and biosensors. Biosens Bioelectron..

[17] Wang F, Liu X, Lu C-H, Willner I (2013). Cysteine-mediated aggregation of Au nanoparticles: the development of a H_2_O_2_ sensor and oxidase-based biosensors. ACS nano.

[18] Eguilaz M, Villalonga R, Yanez-Sedeno P, Pingarron JM (2011). Designing electrochemical interfaces with functionalized magnetic nanoparticles and wrapped carbon nanotubes as platforms for the construction of high-performance bienzyme biosensors. Anal. Chem..

[19] Ramsurn H, Gupta RB (2013). Nanotechnology in solar and biofuels. ACS Sustainable Chem. Eng..

[20] Huang D (2013). Highly sensitive strategy for Hg^2+^ detection in environmental water samples using long lifetime fluorescence quantum dots and gold nanoparticles. Environ. Sci. Technol..

[21] Ben-Sasson M (2014). Surface functionalization of thin-film composite membranes with copper nanoparticles for antimicrobial surface properties. Environ. Sci. Technol..

[22] Petersen EJ (2014). Identification and avoidance of potential artifacts and misinterpretations in nanomaterial ecotoxicity measurements. Environ. Sci. Technol..

[23] Holden P (2013). Ecological nanotoxicology: integrating nanomaterial hazard considerations across the subcellular, population, community, and ecosystems Levels. Acc. Chem. Res..

[24] Corsi I (2014). Common strategies and technologies for the ecosafety assessment and design of nanomaterials entering the marine environment. ACS Nano.

[25] Chaloupka K, Malam Y, Seifalian AM (2010). Nanosilver as a new generation of nanoproduct in biomedical applications. Trends Biotechnol..

[26] Quadros ME, Marr LC (2011). Silver nanoparticles and total aerosols emitted by nanotechnology-related consumer spray products. Environ. Sci. Technol..

[27] Ehdaie B, Krause C, Smith JA (2014). Porous ceramic tablet embedded with silver nanopatches for low-cost point-of-use water purification. Environ. Sci. Technol..

[28] Levard C, Hotze EM, Lowry GV, Brown GE (2012). Environmental transformations of silver nanoparticles: impact on stability and toxicity. Environ. Sci. Technol..

[29] Yu S-j, Yin Y-g, Liu J-f (2013). Silver nanoparticles in the environment. Environ. Sci.: Processes Impacts.

[30] Fabrega J, Renshaw JC, Lead JR (2009). Interactions of silver nanoparticles with *Pseudomonas putida* biofilms. Environ. Sci. Technol..

[31] Xiu ZM, Zhang QB, Puppala HL, Colvin VL, Alvarez PJ (2012). Negligible particle-specific antibacterial activity of silver nanoparticles. Nano Lett..

[32] Piccapietra F, Allue CG, Sigg L, Behra R (2012). Intracellular silver accumulation in *Chlamydomonas reinhardtii* upon exposure to carbonate coated silver nanoparticles and silver nitrate. Environ. Sci. Technol..

[33] Wang J (2013). Phytostimulation of poplars and Arabidopsis exposed to silver nanoparticles and Ag^+^ at sublethal concentrations. Environ. Sci. Technol..

[34] Gray EP (2013). Extraction and analysis of silver and gold nanoparticles from biological tissues using single particle inductively coupled plasma mass spectrometry. Environ. Sci. Technol..

[35] Lee KJ, Browning LM, Nallathamby PD, Osgood CJ, Xu XH (2013). Silver nanoparticles induce developmental stage-specific embryonic phenotypes in zebrafish. Nanoscale.

[36] Osborne OJ (2015). Organ-specific and size-dependent Ag nanoparticle toxicity in gills and intestines of adult zebrafish. ACS Nano.

[37] Wang J, Wang WX (2014). Low bioavailability of silver nanoparticles presents trophic toxicity to marine medaka (*Oryzias melastigma*). Environ. Sci. Technol..

[38] Navarro E, Wagner B, Odzak N, Sigg L, Behra R (2015). Effects of differently coated silver nanoparticles on the photosynthesis of *Chlamydomonas reinhardtii*. Environ. Sci. Technol..

[39] Quigg A (2013). Direct and indirect toxic effects of engineered nanoparticles on algae: role of natural organic matter. ACS Sustainable Chem. Eng..

[40] Chisholm SW (2000). Stirring times in the Southern Ocean. Nature.

[41] Lu Y, Chen W (2012). Sub-nanometre sized metal clusters: from synthetic challenges to the unique property discoveries. Chem. Soc. Rev..

[42] Diez I, Ras RH (2011). Fluorescent silver nanoclusters. Nanoscale.

[43] Shang L, Dong S, Nienhaus GU (2011). Ultra-small fluorescent metal nanoclusters: synthesis and biological applications. Nano Today.

[44] Xu H, Suslick KS (2010). Water-soluble fluorescent silver nanoclusters. Adv. Mater..

[45] Guidez EB, Aikens CM (2012). Theoretical analysis of the optical excitation spectra of silver and gold nanowires. Nanoscale.

[46] Yuan X, Yeow TJ, Zhang Q, Lee JY, Xie J (2012). Highly luminescent Ag^+^ nanoclusters for Hg^2+^ ion detection. Nanoscale.

[47] Zheng K (2015). Boiling water synthesis of ultrastable thiolated silver nanoclusters with aggregation-induced emission. Chem. Commun..

[48] Murray RW (2008). Nanoelectrochemistry: metal nanoparticles, nanoelectrodes, and nanopores. Chem. Rev..

[49] Zheng K, Yuan X, Goswami N, Zhang Q, Xie J (2014). Recent advances in the synthesis, characterization, and biomedical applications of ultrasmall thiolated silver nanoclusters. RSC Adv..

[50] Zheng K, Setyawati MI, Lim TP, Leong DT, Xie J (2016). Antimicrobial cluster bombs: silver nanoclusters packed with daptomycin. ACS Nano.

[51] Yuan X, Setyawati MI, Leong DT, Xie J (2013). Ultrasmall Ag^+^-rich nanoclusters as highly efficient nanoreservoirs for bacterial killing. Nano Res..

[52] Yeh HC, Sharma J, Han JJ, Martinez JS, Werner JH (2010). A DNA-silver nanocluster probe that fluoresces upon hybridization. Nano Lett..

[53] Qi L (2015). Fluorescent DNA-protected silver nanoclusters for ligand-HIV RNA interaction assay. Anal. Chem..

[54] Zhu J (2015). G-quadruplex enhanced fluorescence of DNA-silver nanoclusters and their application in bioimaging. Nanoscale.

[55] Gupta GS (2017). Laboratory scale microbial food chain to study bioaccumulation, biomagnification, and ecotoxicity of cadmium telluride quantum dots. Environ. Sci. Technol..

[56] Cronholm P (2013). Intracellular uptake and toxicity of Ag and CuO nanoparticles: a comparison between nanoparticles and their corresponding metal ions. Small.

[57] Gao J, Lin L, Wei A, Sepúlveda MS (2017). Protein corona analysis of silver nanoparticles exposed to fish plasma. Environ. Sci. Technol. Lett..

[58] Lowry GV, Gregory KB, Apte SC, Lead JR (2012). Transformations of nanomaterials in the environment. Environ. Sci. Technol..

[59] Zhang B (2017). Metabolic responses of the growing Daphnia similis to chronic AgNPs exposure as revealed by GC-Q-TOF/MS and LC-Q-TOF/MS. Water Res..

[60] Du C (2017). Biological effect of aqueous C_60_ aggregates on Scenedesmus obliquus revealed by transcriptomics and non-targeted metabolomics. J. Hazard Mater..

[61] Safar R (2015). Human monocyte response to *S*-nitrosoglutathione-loaded nanoparticles: uptake, viability, and transcriptome. Mol. Pharm..

[62] Leclerc S, Wilkinson KJ (2014). Bioaccumulation of nanosilver by *Chlamydomonas reinhardtii*-nanoparticle or the free ion?. Environ. Sci. Technol..

[63] Chen C (2015). Toxicogenomic responses of the model legume *Medicago truncatula* to aged biosolids containing a mixture of nanomaterials (TiO_2_, Ag, and ZnO) from a pilot wastewater treatment plant. Environ. Sci. Technol..

[64] Sun D (2017). Transcriptome analysis reveals silver nanoparticle-decorated quercetin antibacterial molecular mechanism. ACS Appl. Mater. Interfaces.

[65] Poynton HC (2012). Toxicogenomic responses of nanotoxicity in *Daphnia magna* exposed to silver nitrate and coated silver nanoparticles. Environ. Sci. Technol..

[66] Yuan X (2013). Highly luminescent silver nanoclusters with tunable emissions: cyclic reduction–decomposition synthesis and antimicrobial properties. NPG Asia Mater..

[67] Yuan X (2013). Glutathione-protected silver nanoclusters as cysteine-selective fluorometric and colorimetric probe. Anal. Chem..

[68] Xu FF, Imlay JA (2012). Silver(I), mercury(II), cadmium(II), and zinc(II) target exposed enzymic iron-sulfur clusters when they toxify Escherichia coli. Appl. Environ. Microbiol..

[69] Zhang L (2016). Uptake and effect of highly fluorescent silver nanoclusters on *Scenedesmus obliquus*. Chemosphere.

[70] Kaveh R (2013). Changes in *Arabidopsis thaliana* gene expression in response to silver nanoparticles and silver ions. Environ. Sci. Technol..

[71] Pillai S (2014). Linking toxicity and adaptive responses across the transcriptome, proteome, and phenotype of *Chlamydomonas reinhardtii* exposed to silver. Proc. Nati. Acad. Sci. USA.

[72] Diez I (2011). Functionalization of nanofibrillated cellulose with silver nanoclusters: fluorescence and antibacterial activity. Macromol. Biosci..

[73] Wang L (2015). Use of synchrotron radiation-analytical techniques to reveal chemical origin of silver-nanoparticle cytotoxicity. ACS Nano.

[74] Kanehisa M, Sato Y, Kawashima M, Furumichi M, Tanabe M (2016). KEGG as a reference resource for gene and protein annotation. Nucleic Acids Res..

