# Supplementary material for: Unraveling the molecular mechanism of photosynthetic toxicity of highly fluorescent silver nanoclusters to Scenedesmus obliquus
Source: Sci Rep. 2017 Nov 27;7:16432. doi: 10.1038/s41598-017-16634-5 (PMC5703894; doi:10.1038/s41598-017-16634-5)
Supplement: Supplementary file 1 — Supplementary Information [file 41598_2017_16634_MOESM1_ESM.doc]

**Supplementary Information**

**Unraveling the molecular mechanism of photosynthetic toxicity of highly fluorescent silver nanoclusters to *Scenedesmus obliquus***

**Li Zhang**^1^, **Nirmal Goswami**^2^, **Jianping Xie**^2^, **Bo Zhang**^1^, **Yiliang He**^1^,*****

^1^School of Environmental Science and Engineering, Shanghai Jiao Tong University, No. 800 Dongchuan Road, Minhang District, Shanghai, 200240, China

^2^Department of Chemical and Biomolecular Engineering , National University of Singapore, 4 Engineering Drive 4, #03-18,117585, Singapore

*****Corresponding author:

Dr. Yiliang He

School of Environmental Science & Engineering, Shanghai Jiao Tong University, Shanghai, 200240, China

Tel: +86 21 54744008; Fax: +86 21 54740825

E-mail : [ylhe@sjtu.edu.cn](mailto:ylhe@sjtu.edu.cn)

**Supplementary Experimental Methods**

**Algae culture and growth conditions.** *Scenedesmus* *obliquus* (No.FACHB-417) was cultured according to the following procedure. A small volume of algal cells was transferred into 1L of SE culture medium (Supplementary Table S1 ) to obtain an initial cell density of ～5×10^5^ cells mL^-1^, and then pre-cultured until the exponential growth phase of was reached (typically 4～5d). Algae were collected by filtration using 0.22μm cellulose membrane and resuspended in SE culture medium in order to obtain the experimental solutions (5～10×10^5^ cells mL^-1^). Algae were cultured in artificial climate chamber under a standard lighting and temperature condition (12 h light/12 h dark photoperiod; illumination 2500～3000lx; 25 ^o^C; artificial shaking three times every day ) **according to the guidelines of** the Institute of Hydrobiology of Chinese Academy of Sciences. Cell numbers were measured using linear regression relationship (y=3134x-12, R^2^=0.9946) between the number of algal cells (×10^4^ cells mL^-1^) counted by [blood cell counting chamber](https://www.baidu.com/link?url=LVwPLR6hkMS-_vDlMwaALD6Pm_VGq-yQ8c23BNQL29juK8AJNaomM6kzjDNJwZ0kGha7W4joztJWSnYkz8HHI8PVI9BbxnA3XyHlCySQ9K4NDHQLGzUpYAAal0EhxAstQ3Obg01zKbykPSdy3rQsn_&wd=&eqid=d08989e30003848e0000000358778f21) and their OD_688_ (Supplementary Fig. S1).

**Synthesis of highly fluorescent r-Ag NCs.** Highly fluorescent r-Ag NCs were freshly synthesized according to previous studies^1, 2^. Briefly, under vigorous stirring, the aqueous solutions of GSH (1.5mL, 50 mM) and NaBH_4_ (0.5mL, 112 mM) were firstly mixed in Milli-Q water (48.5mL) at room temperature, followed by the addition of an aqueous solution of AgNO_3_ (1.25mL, 20 mM) to form thiolate-Ag^I^ complexes. After 5 min, a deep-red solution of Ag NCs was obtained and then incubated at room temperature without stirring for about 6h. This deep-red solution was unstable and gradually decomposed to colorless. After that, in the process of vigorous stirring, the same amount of NaBH_4_ (0.5mL, 112 mM, drop by drop) was introduced into this colorless solution at room temperature, and a light-brown Ag NCs solution was brought about after 30 min. Finally, without stirring, this light-brown Ag NC solution was further incubated at room temperature for about 8 h, and a strong red fluorescence was then observed from r-Ag NCs in the aqueous phase. The r-Ag NCs were collected, purified by using dialysis tubing with a molecular weight cutoff (MWCO) of 3 kDa for 3~4 h, and stored at 4 ^o^C before usage.

**Dissolution of r-Ag NCs.** To assess the dissolution of r-Ag NCs in SE culture medium, we determined the concentrations of Ag^+^ ions originating from the r-Ag NCs. Briefly, three beakers were set up with 2L of SE culture medium, a final concentration of 135μg L^-1^ r-AgNCs diluted with SE medium was added into each dialysis tubing with a molecular weight cutoff (MWCO) of 3 kDa (**Beijing** Solarbio Science﹠Technology Co., Ltd) and subjected to dialysis in SE medium at 4 ^o^C. The SE culture medium of each treatment replicate containing Ag^+^ ions was sampled at 2h、6h、12h、24h、48h、72h、96h and 120h, acidified with 2% of nitric acid, and further measured by inductively coupled plasma mass spectrometry (ICP-MS, Agilent 7500a, USA). The assessment of the dissolution of r-Ag NCs in Milli-Q water was also performed with the same method mentioned above.

**Intracellular uptake of r- Ag NCs using TEM and LSCM.** The TEM samples of algae cells exposed to all the silver treatment were prepared according to the previously reported protocol with slight modification^3^. Refer to the second scheme of algae photosynthetic toxicity. After 96h exposure, algal cells from the control, the 135μg L^-1^ r-AgNCs treatment, the 135μg L^-1^ r-AgNCs (contained 0.5 mM of L-cysteine) treatment and the 10μg L^-1^ silver ions treatment were collected and then washed thrice with ultrapure water. Thereafter, algal cells were firstly fixed with 2.5% glutaraldehyde at 4 ^o^C overnight and rinsed thrice with the phosphate buffer (pH7.0) , and then were post-fixed with 1% OsO_4_ at 4 ^o^C for 5 h and washed thrice with the phosphate buffer (pH7.0). Furthermore, cells were dehydrated by a series of ethanol (50%, 70%, 90%), 1:1mixture of ethanol (90%) and acetone (90%), acetone (90%) and acetone (100%) at 4 ^o^C for 15 min at each step. After dehydration, cells were immersed in 1:1 and 1:2 mixtures of acetone and ethoxyline resin for 1 h and 4 h, respectively, transferred to ethoxyline resin at room temperature overnight, and were hardened at 60 ^o^C for 48 h. Finally, sections (70nm) of the samples were cut and stained with uranyl acetate and alkaline lead citrate, and were observed under biology TEM (FEI, Tecnai G2 spirit Biotwin operated at 120kV).

Similar to the preparation of TEM samples, algal cells from four treatments were also collected after 96h exposure, resuspended in SE medium, and were further observed under LSCM (Leica TCS SP5) using laser of 458nm.

**Library construction and RNA sequencing.** Total RNA of algae cells was extracted by using Trizol Reagent according to the manufacturer’s instructions (Invitrogen, USA). The concentration and integrity of RNA were determined with a Nanodrop 2000 UV-Vis Spectrophotometer (Nanodrop, USA) and agarose gel electrophoresis, respectively. The mRNA was isolated from total RNA using magnetic beads with oligo-dT attached to the poly-A of the mRNA. The purified mRNA was then chopped into small fragment of 200-300bp with fragmentation buffer. Subsequently, the fragments were used to synthesize first-strand cDNA using random primers and reverse transcriptase, and first-strand cDNA was transformed into double-strand cDNA with RNase H and DNA polymerase I. The protruding terminus of the DNA fragments were end-repaired under the function of 3 '- 5' exonuclease and polymerase. The end-repaired DNA fragments were ligated with sequencing adapters through the complementary action between the base “A” of the 3’ end of DNA fragments and the base “T” of 3’ end of the adapters. After the unsuitable fragments were removed by using AMPure XP beads (Beckman Coulter, Shanghai, China), the sequencing library was constructed using polymerase chain reaction (PCR) amplification, and further checked with PicoGreen and spectrophotometry and quantified with Agilent 2100 Bioanalyzer. The multiplexed DNA libraries were normalized to a 10 nM and further mixed in term of equal volume. Finally, the sequencing library was gradually diluted and quantified to 4～5 pM and sequenced on the Illumina NextSeq 500 platform.

**Real-time reverse transcription PCR (qRT-PCR).** Four target genes in light reaction of photosynthesis and a target gene in photosynthetic carbon reduction cycle were selected from differentially expressed genes in the transcriptome data for qRT-PCR analysis. Briefly, total RNA were extracted from algae cells using the same protocols as described above for the RNA sequencing. cDNA was synthesized by using RNase H and DNA polymerase I. The qRT-PCR was carried out in a 20μL reaction mixture containing 10μL 2×SYBR real-time PCR premixture, 0.5μL each primer(10μM),1μL diluted cDNA mix and 8μL R-Nase free water. The primer sequences for qRT-PCR analysis are presented in the Supplementary Table S5. The qRT-PCR reactions were performed on a StepOne Plus qRT-PCR System (Applied Biosystems) with the following program: 95 ^o^C for 5min, 40 cycles of 95 ^o^C for 15s, 60 ^o^C for 30s. All samples were done in triplicates for each target gene and negative controls were also performed by operating the qRT-PCR reactions without reverse transcriptase. The gene expression levels were calculated by using the cycle threshold (Ct) values after the qRT-PCR data was normalized with the reference gene (α-tubulin).

**Supplementary Figures and Tables**

**Table S1.** The composition of **Bristol’s solution** (SE culture medium) . **SE** medium were sterilized in autoclave instrument (121 ^o^C, 30min) and used for further experiments.

| **No.** | **Ingredient** | **Concentration of stock solution** | **Usage** |
| --- | --- | --- | --- |
| **1** | NaNO_3_ | 25g/100mL of ultrapure water | 1mL/L |
| **2** | K_2_HPO_4_ | 7.5g/100mL of ultrapure water | 1mL/L |
| **3** | MgSO_4_·7H_2_O | 7.5g/100mL of ultrapure water | 1mL/L |
| **4** | CaCl_2_·2H_2_O | 2.5g/100mL of ultrapure water | 1mL/L |
| **5** | KH_2_PO_4_ | 17.5g/100mL of ultrapure water | 1mL/L |
| **6** | NaCl | 2.5g/100mL of ultrapure water | 1mL/L |
| **7** | FeCl3·6H_2_O | 0.5g/100mL of ultrapure water | 1mL/L |
| **8** | EDTA-Fe | ① 4.1 mL of concentrated hydrochloric acid was diluted to 50 mL of ultrapure water (solution one);  ② 0.9306g of EDTA-Na_2_ was dissolved into 50 mL of ultrapure water (solution two);  ③ 0.901g of FeCl_3_·6H_2_O was dissolved into 10 mL of the solution one (solution three);  ④ 10 mL of the solution two was added into the solution three (solution four);  ⑤ The solution four was then diluted to 1L of ultrapure water (stock solution). | 1mL/L |
| **9** | H_3_BO_3_  MnCl_2_·4H_2_O  ZnSO_4_·7H_2_O  Na_2_MoO_4_·2H_2_O  CuSO_4_·5H_2_O  Co(NO_3_)_2_·6H_2_O | 2.86g/100mL of ultrapure water  1.86g/100mL of ultrapure water  0.22g/100mL of ultrapure water  0.39g/100mL of ultrapure water  0.08g/100mL of ultrapure water  0.05g/100mL of ultrapure water | 1mL/L |
| **10** |  | ① 200g of garden soil with fertilizer were dissolved  into l L of ultrapure water ;  ② The mixture was heated in boiling water for 3h,  cooled down , and was deposited for 24h;  ③ The second step was done in triplicate, and the  mixture was then filtered to obtain the supernatant;  ④The supernatant was sterilized in a SANYO  autoclave (121 ^o^C, 30 min) and stored at 4 ^o^C  before usage. | 40mL/L |

**
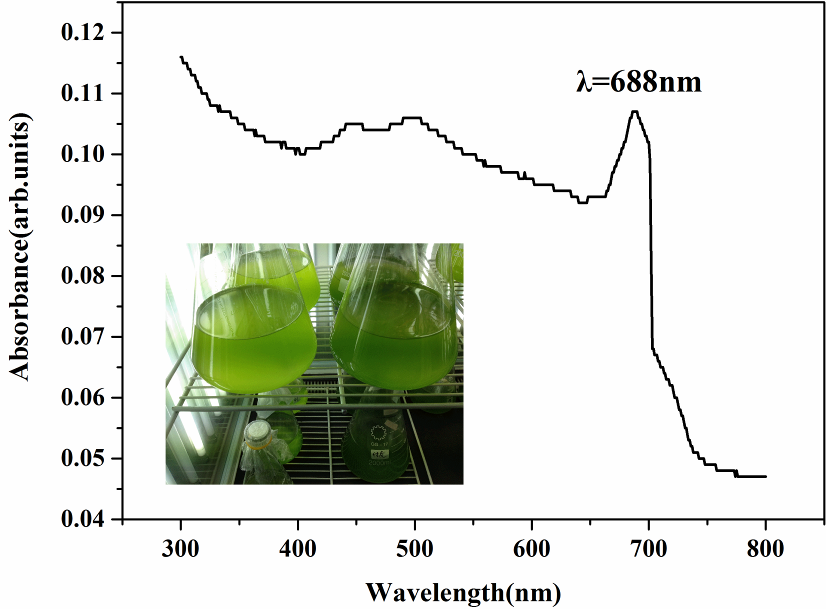
**

**Figure S1.** The UV-Vis spectra determined by a Agilent UV-7500 spectrometer and photographs (inset) of the *Scenedesmus* *obliquus.*

**
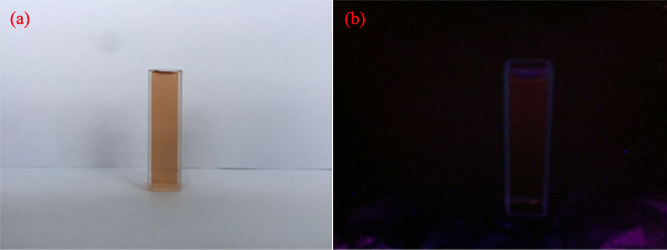
**

**Figure S2.** The photographs of r-Ag NCs in water under visible (a) and UV (b, λ = 365nm) light. The right photograph of r-Ag NCs was taken under dark surroundings.

**
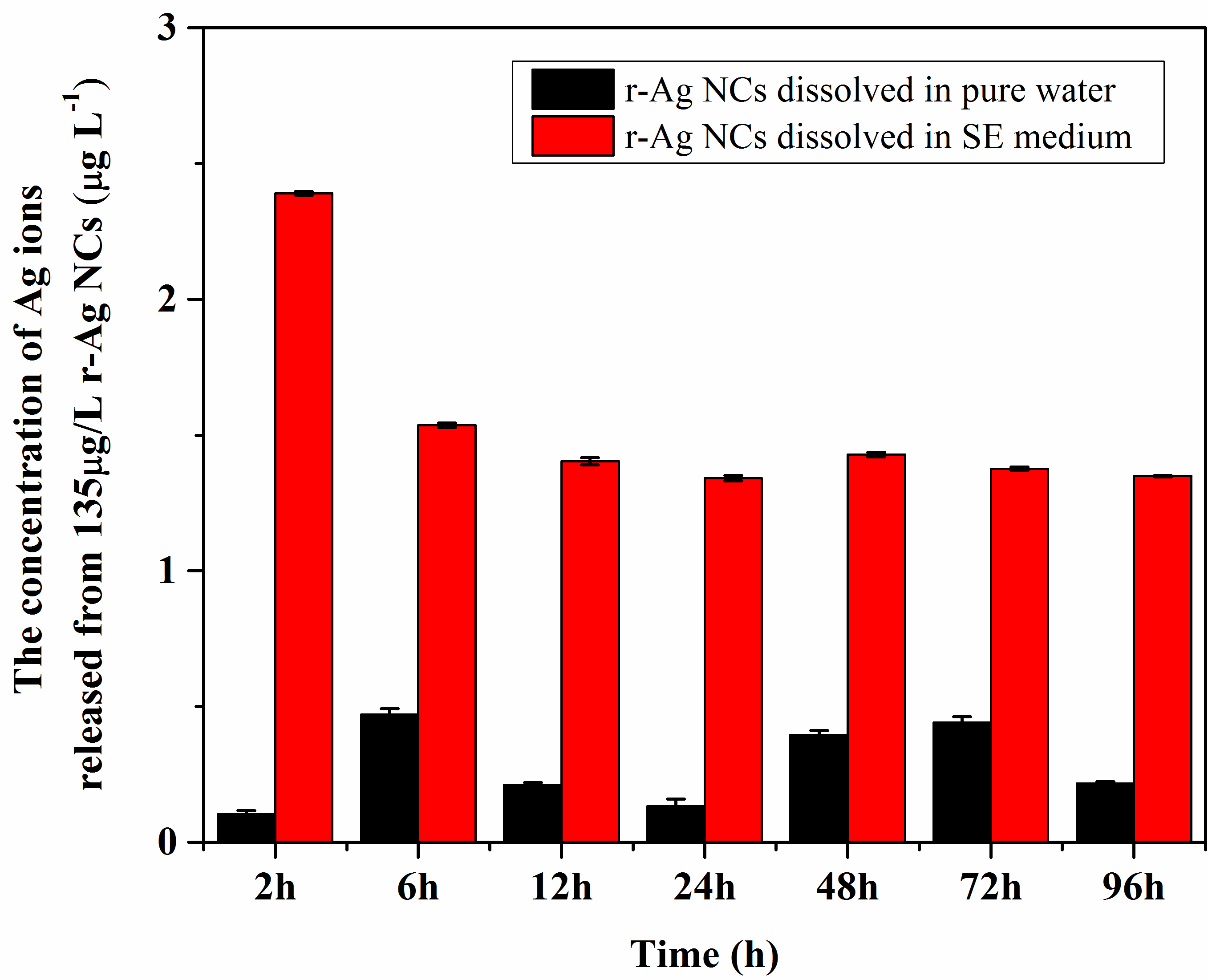
**

**Figure S3.** The concentration of Ag^+^ released from 135μg L^-1^ r-Ag NCs in pure water and in SE medium.


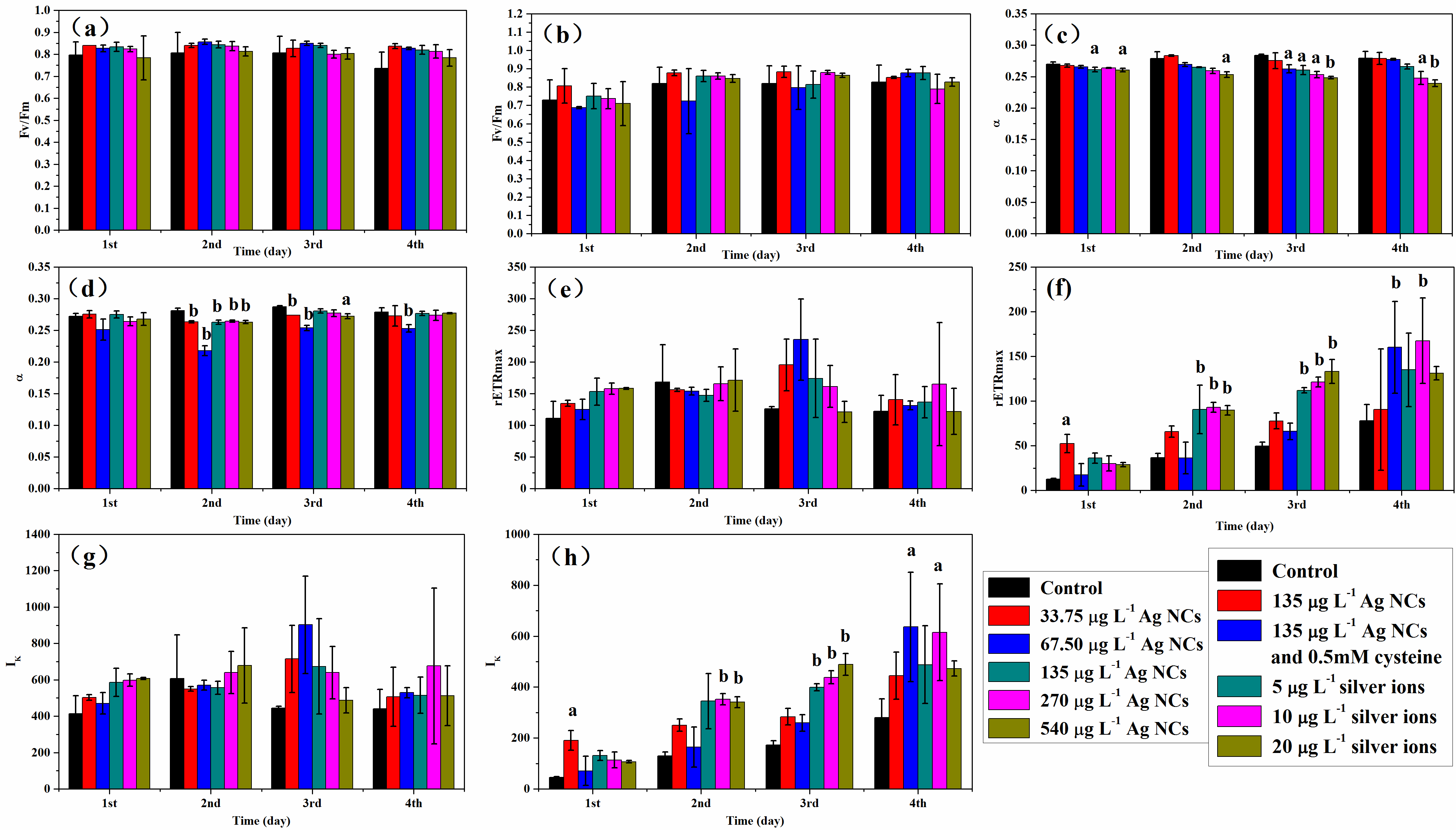


**Figure S4.** The F_v_/F_m_, rETR_Max_, α and I_k_ of algae cells to various concentrations of r-Ag NCs (a、c、e and g) and Ag ions (b、d、f and h) for the 24-96h. Controls were nonexposed algae cells growing in SE medium. Error bars represent the standard deviations for technical triplicates. Letters refer to significant differences identified by performing a one-way analysis of variance (ANOVA) followed by independent t-test. (^a^ *P* < 0.05; ^b^ *P* < 0.01).

**
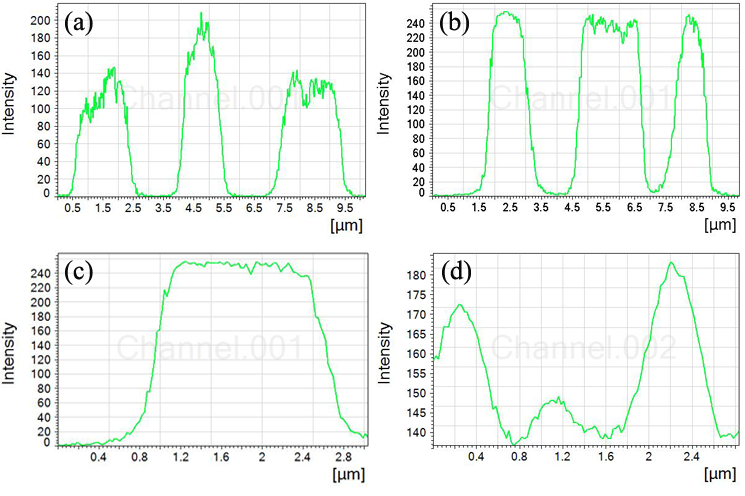
**

**Figure S5.** The fluorescence intensity at 458nm excitation of designated spots of algae cells exposed to r- Ag NCs or AgNO_3_. (a) Control. (b) r-Ag NCs. (c) r-Ag NCs + L- cysteine. (d) AgNO_3_.


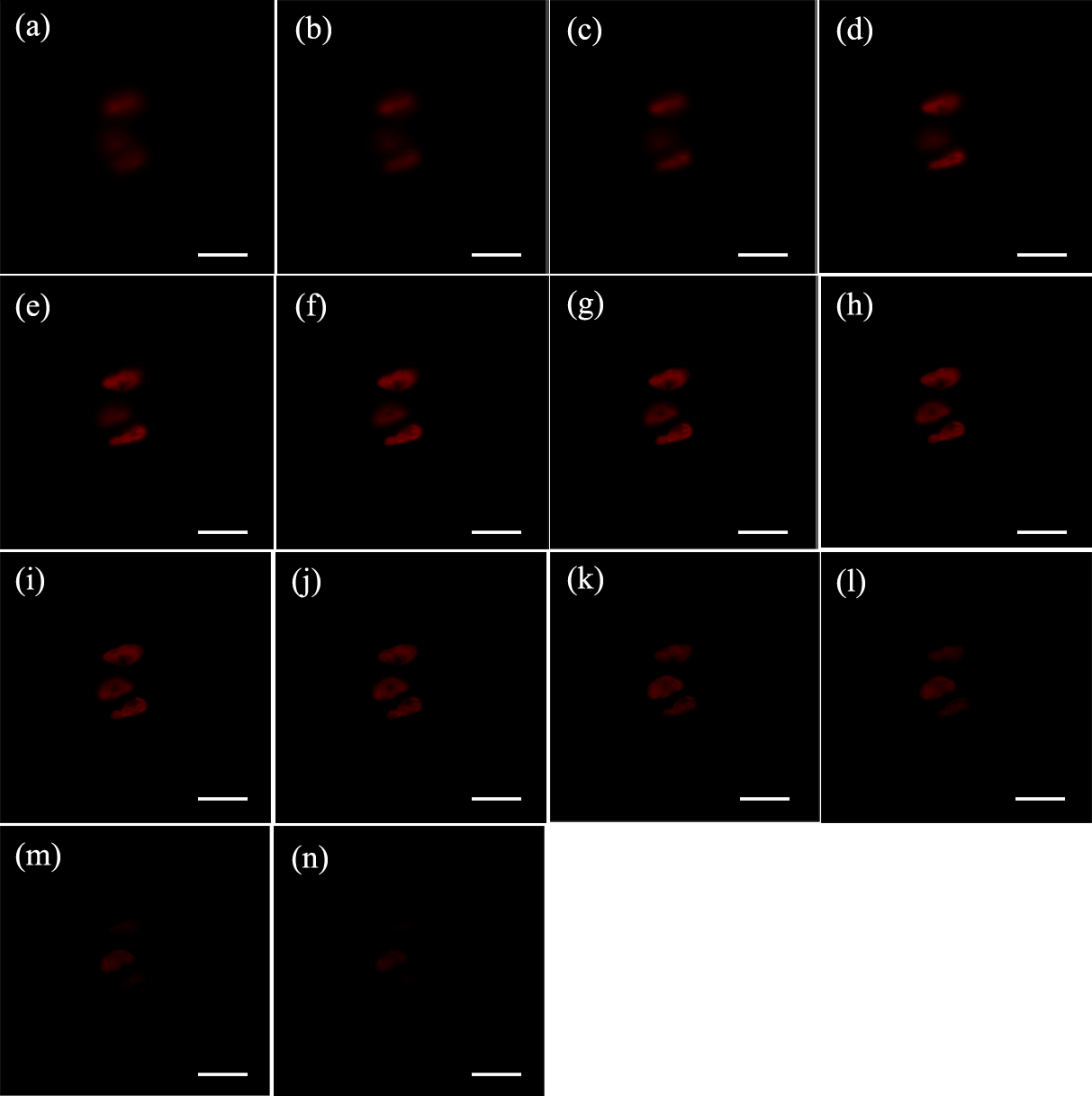


**Figure S6.** The Z-axis images of the algae cells exposed to r-Ag NCs (without L-cysteine) from the top to bottom of the sample as evidence for the r-Ag NCs internalization. Excitation wavelength was 458 nm. The scale bar is 2.5 μm.

**
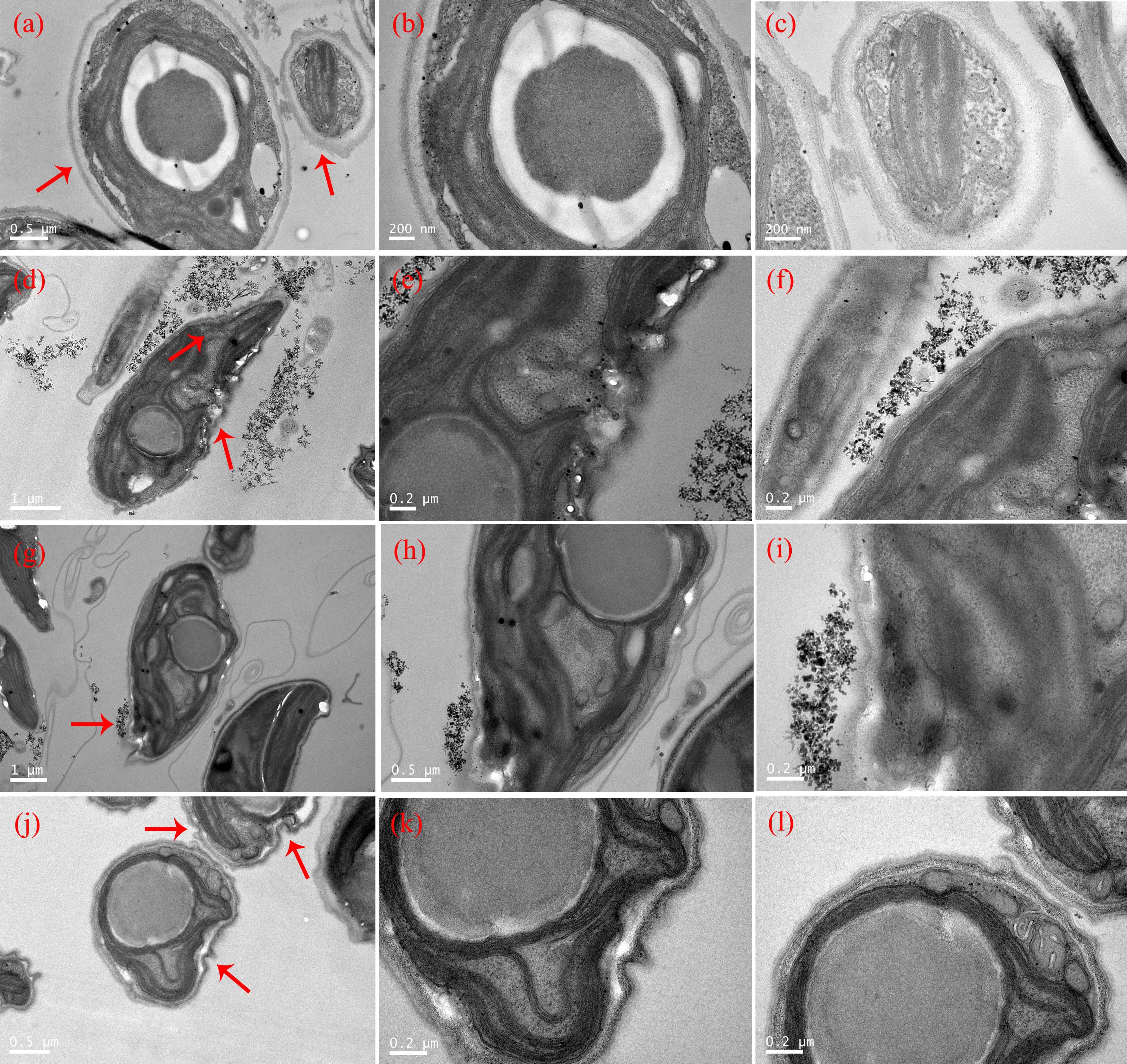
**

**Figure S7.** The TEM images of algae cells exposed for 96h to r-Ag NCs or AgNO_3_. (a) control. (d) r-Ag NCs without L-cysteine. (g) r-Ag NCs with L-cysteine. (j) AgNO_3_. b, c, e, f, h, i, k and l are the magnified images (a), (d), (g) and (j), respectively.

**Table S2.** The total RNA was checked using NanoDrop 2000 UV-Vis Spectrophotometer.

| **Swimming**  **Lane**  **(No.)** | **Samples**  **(No.)** | **Concentration**  **(ng/μL)** | **260/280** | **260/230** | **Volume**  **(μL)** | **Total RNA**  **(μg)** |
| --- | --- | --- | --- | --- | --- | --- |
| 1 | A1 | 505.3 | 1.98 | 2.34 | 45 | 22.74 |
| 2 | A2 | 450.8 | 1.98 | 2.37 | 45 | 20.29 |
| 3 | A3 | 556.1 | 1.98 | 2.39 | 45 | 25.02 |
| 4 | A4 | 497.2 | 1.98 | 2.47 | 45 | 22.37 |
| 5 | B1 | 574.3 | 1.98 | 2.43 | 45 | 25.84 |
| 6 | B2 | 1631.3 | 1.18 | 1.18 | 45 | 73.41 |
| 7 | B3 | 484.1 | 1.96 | 2.49 | 45 | 21.78 |
| 8 | B4 | 340.4 | 1.98 | 2.50 | 45 | 15.32 |
| 9 | C1 | 892.5 | 1.57 | 1.60 | 45 | 40.16 |
| 10 | C2 | 486.5 | 1.94 | 2.39 | 45 | 21.89 |
| 11 | C3 | 578.7 | 1.95 | 0.89 | 45 | 26.04 |
| 12 | C4 | 271.8 | 1.84 | 2.22 | 45 | 12.23 |
| 13 | D1 | 424.7 | 11.88 | 2.32 | 45 | 19.11 |
| 14 | D2 | 449.2 | 1.94 | 2.48 | 45 | 20.21 |
| 15 | D3 | 443.0 | 1.90 | 2.41 | 45 | 19.94 |
| 16 | D4 | 389.0 | 1.94 | 2.47 | 45 | 17.51 |

Note: The samples number of A, B, C and D represented the control, the 135μg L^-1^ r-AgNCs treatment, the 135μg L^-1^ r-AgNCs (contained 0.5 mM of L-cysteine) treatment and the 10μg L^-1^ silver ions treatment, respectively. Each treatment was done in biological quadruplicates.

**
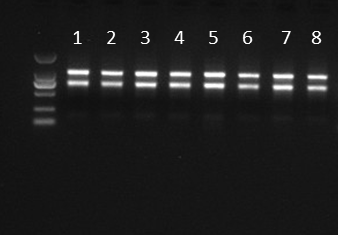

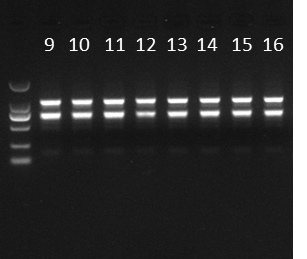

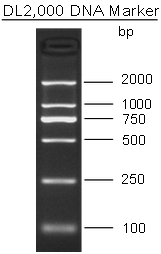
**

**Figure S8.** The total RNA was checked by agarose gel electrophoresis. The swimming lane number (1～16) represented the control (No.1～4), the 135μg L^-1^ r-AgNCs treatment (No.5～8), the 135μg L^-1^ r-AgNCs (contained 0.5 mM of L-cysteine) treatment (No.9～12) and the 10μg L^-1^ silver ions treatment (No.13～16), respectively. Each treatment were done in biological quadruplicates.

**Table S3.** Summary of the Trinity *de novo* assembly of transcriptomic profiles of *Scenedesmus* *obliquus* exposed for 96h to various concentration of r-Ag NCs and Ag^+^ .

| Raw Sequencing Reads | | | | | | | |
| --- | --- | --- | --- | --- | --- | --- | --- |
| **Samples** | Reads | Raw Reads | Raw Data (bp) | Q30(%) | Q20(%) | GC% | |
| **A** | Paired | 69,344,652 | 9,784,168,658 | 81.51 | 89.72 | 60.80 | |
| **B** | Paired | 55,782,342 | 7,999,097,071 | 79.11 | 88.91 | 59.83 | |
| **C** | Paired | 67,783,242 | 9,611,213,918 | 79.49 | 89.08 | 60.08 | |
| **D** | Paired | 51,227,222 | 7,358,295,460 | 78.90 | 88.64 | 59.94 | |
| High Quality Reads | | | | | | | |
| **Samples** | Reads | Clean Reads | Clean Data (bp) | Useful Reads% | Useful Data % |  | |
| **A** | Paired | 55,164,844 | 7,444,330,932 | 79.55 | 76.09 |  | |
| **B** | Paired | 45,314,040 | 6,069,264,166 | 81.23 | 75.87 |  | |
| **C** | Paired | 55,271,570 | 7,366,979,037 | 81.54 | 76.65 |  | |
| **D** | Paired | 41,178,254 | 5,566,183,124 | 80.38 | 75.65 |  | |
| Sequence Assembly Statistic | | | | | | | |
|  | Total Length (bp) | Sequence No. | Max. Length (bp) | Ave. Length (bp) | N50 | > N50 Reads No. | GC% |
| **Contig** | 98,885,547 | 256,501 | 37,005 | 385.52 | 587 | 34,689 | 57.66 |
| **Transcript** | 93,207,922 | 140,497 | 31,423 | 663 | 995 | 22,607 | 57.80 |
| **Unigene** | 31,358,003 | 26,570 | 31,423 | 1,180 | 1,914 | 4,604 | 58.52 |

Note: (1) The samples number of A, B, C and D represented the control, the 135μg L^-1^ r-AgNCs (without 0.5 mM of L-cysteine) treatment, the 135μg L^-1^ r-AgNCs (with 0.5 mM of L-cysteine) treatment and the 10μg L^-1^ silver ions treatment, respectively. (2) Q20: percentage is the proportion of nucleotides with a quality value > 20 in raw sequencing reads. Q30: percentage is the proportion of nucleotides with a quality value > 30 in raw sequencing reads. N50: unigene length-weighted median. GC %: percentage of G and C bases.

**
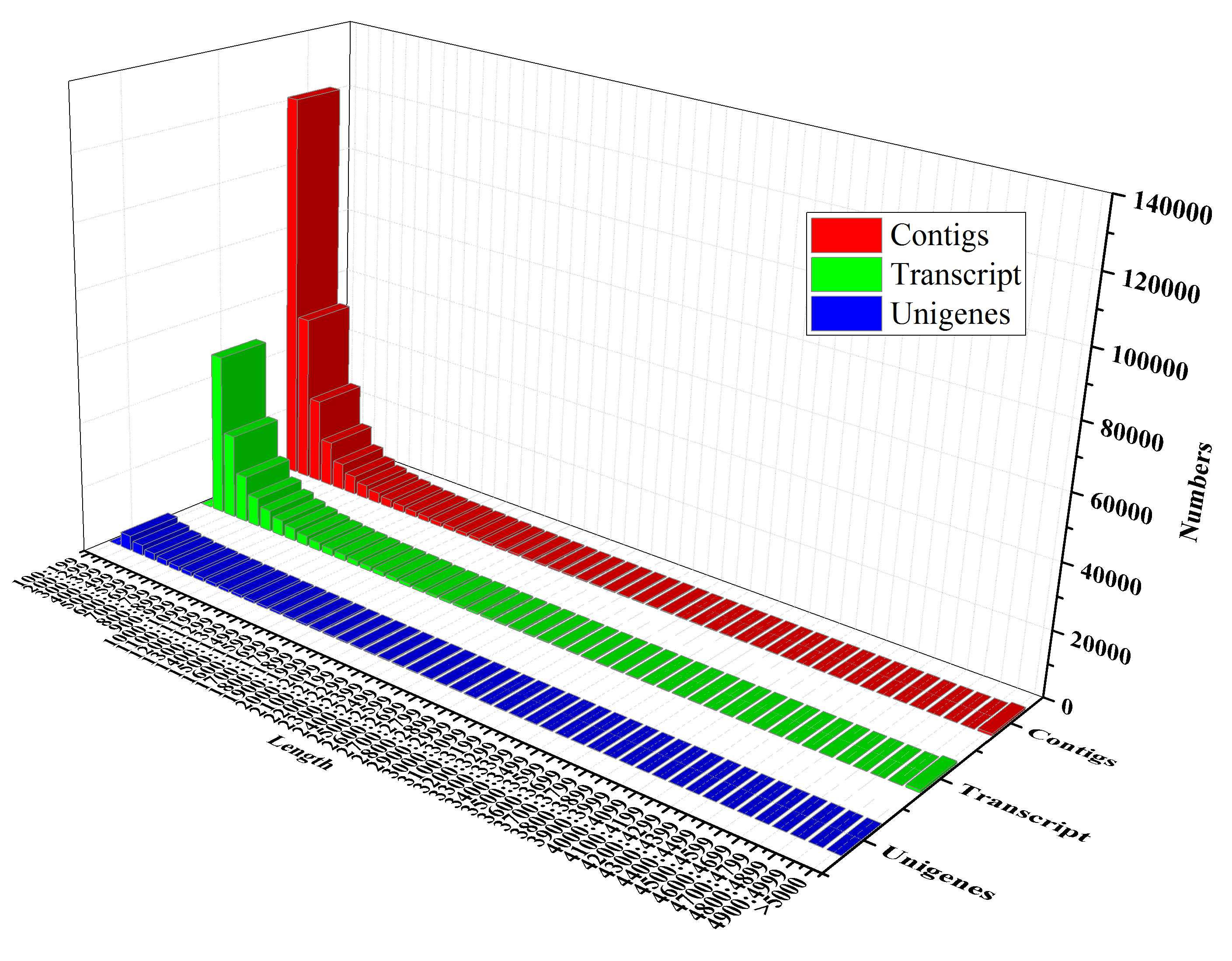
**

**Figure S9.** Histogram of length distribution of contiges (a), transcripts (b) and unigenes (c), with the number of contiges, transcripts and unigenes on the y-axis and the length distribution on the x-axis.


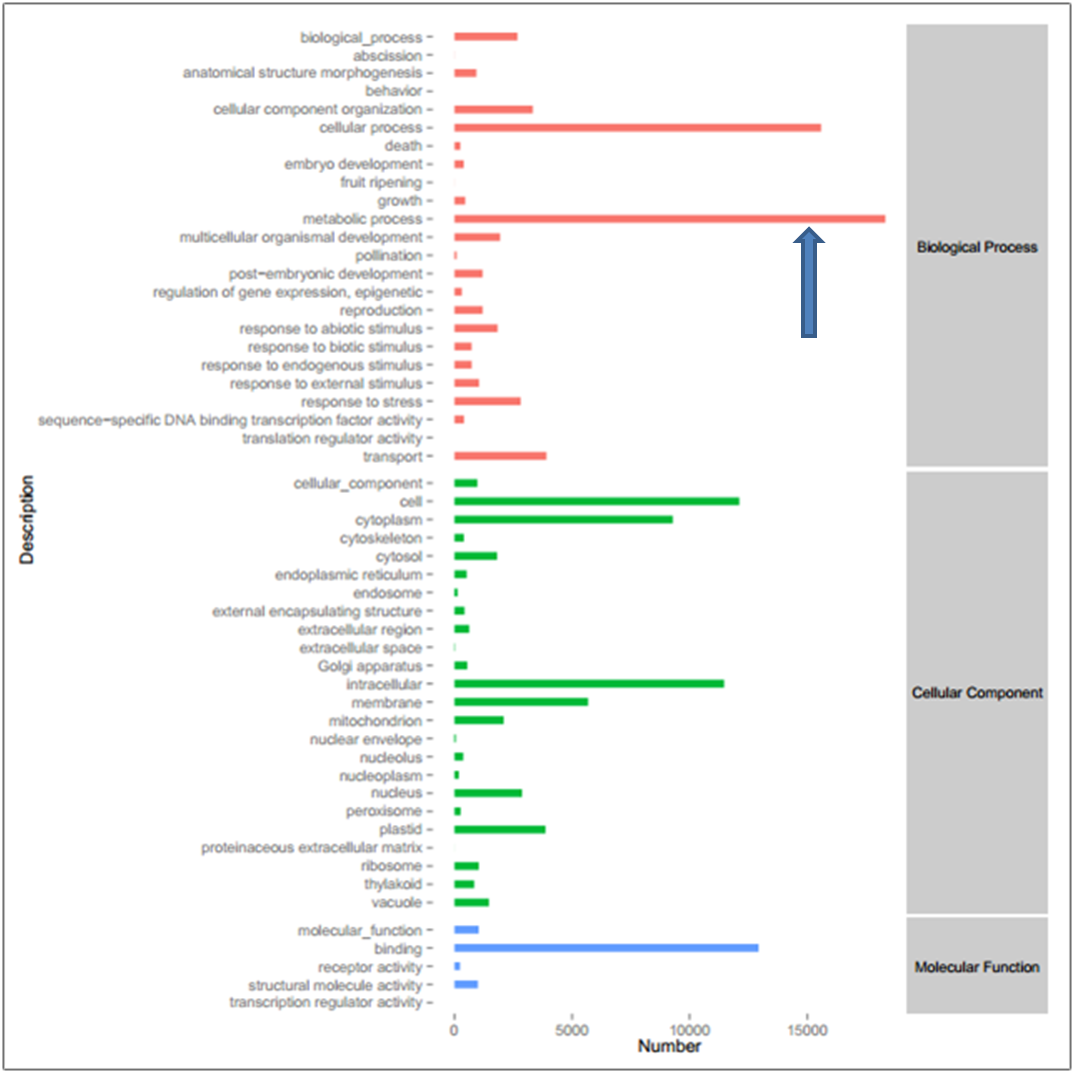


**Figure S10.** The Gene Ontology (GO) annotations of unigenes. The bars represent the number of the unigenes (x-axis) in different GO terms (y-axis).


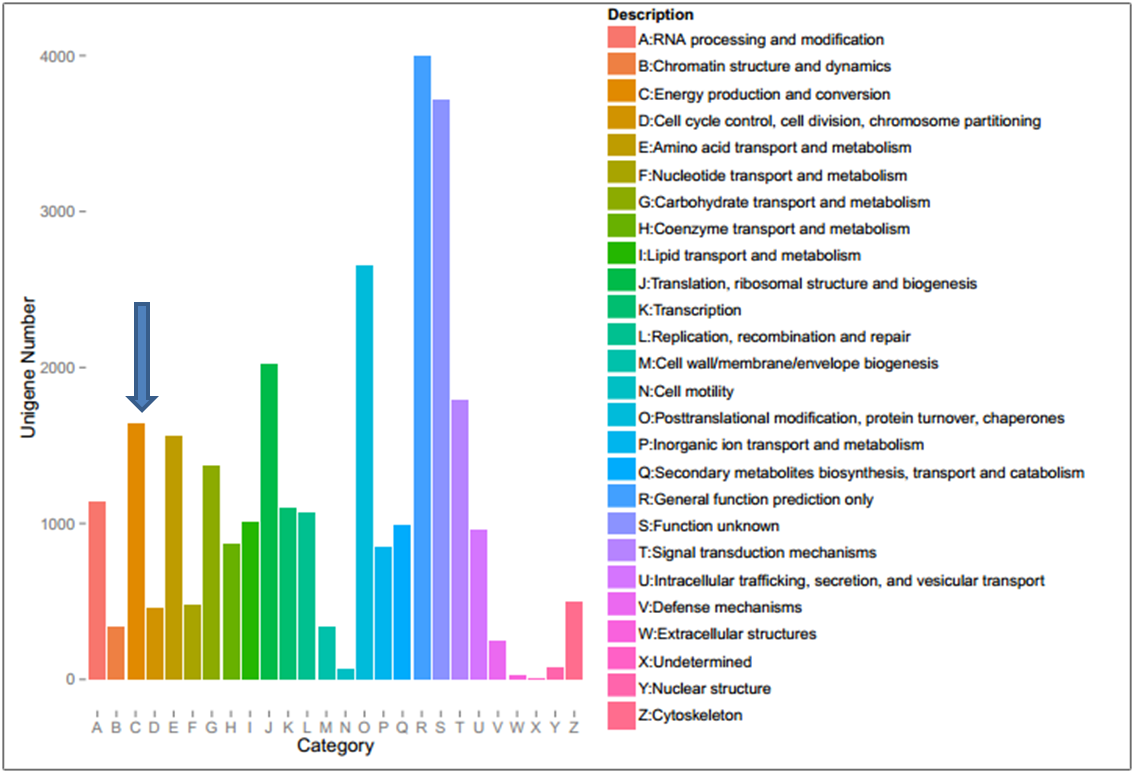


**Figure S11.** The Evolutionary genealogy of genes: Non-supervised Orthologous Groups (eggNOG) annotations of unigenes. The bars represent the number of the unigenes (y-axis) in different eggNOG categories (x-axis).


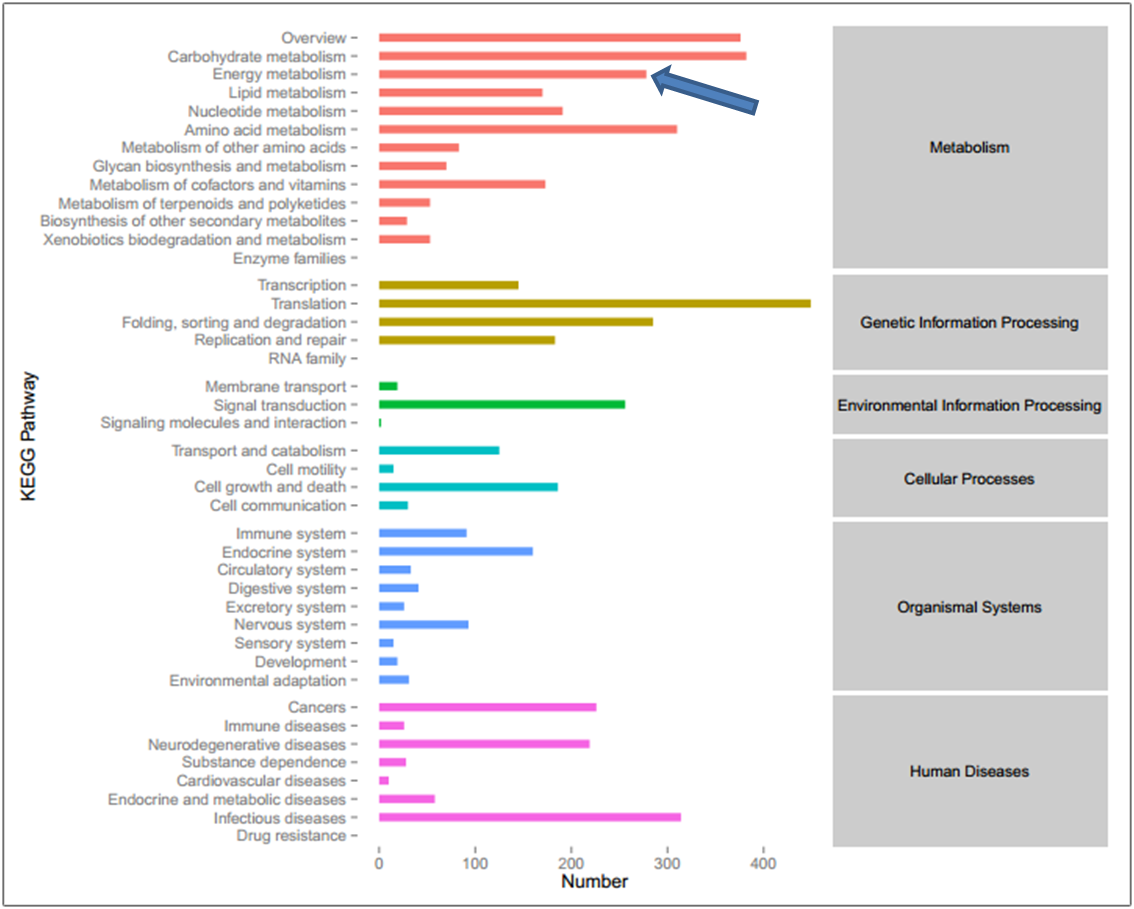


**Figure S12.** The Kyoto Encyclopedia of Genes and Genomes (KEGG) annotations of unigenes. The bars represent the number of the unigenes (x-axis) in different KEGG metabolic pathways (y-axis).


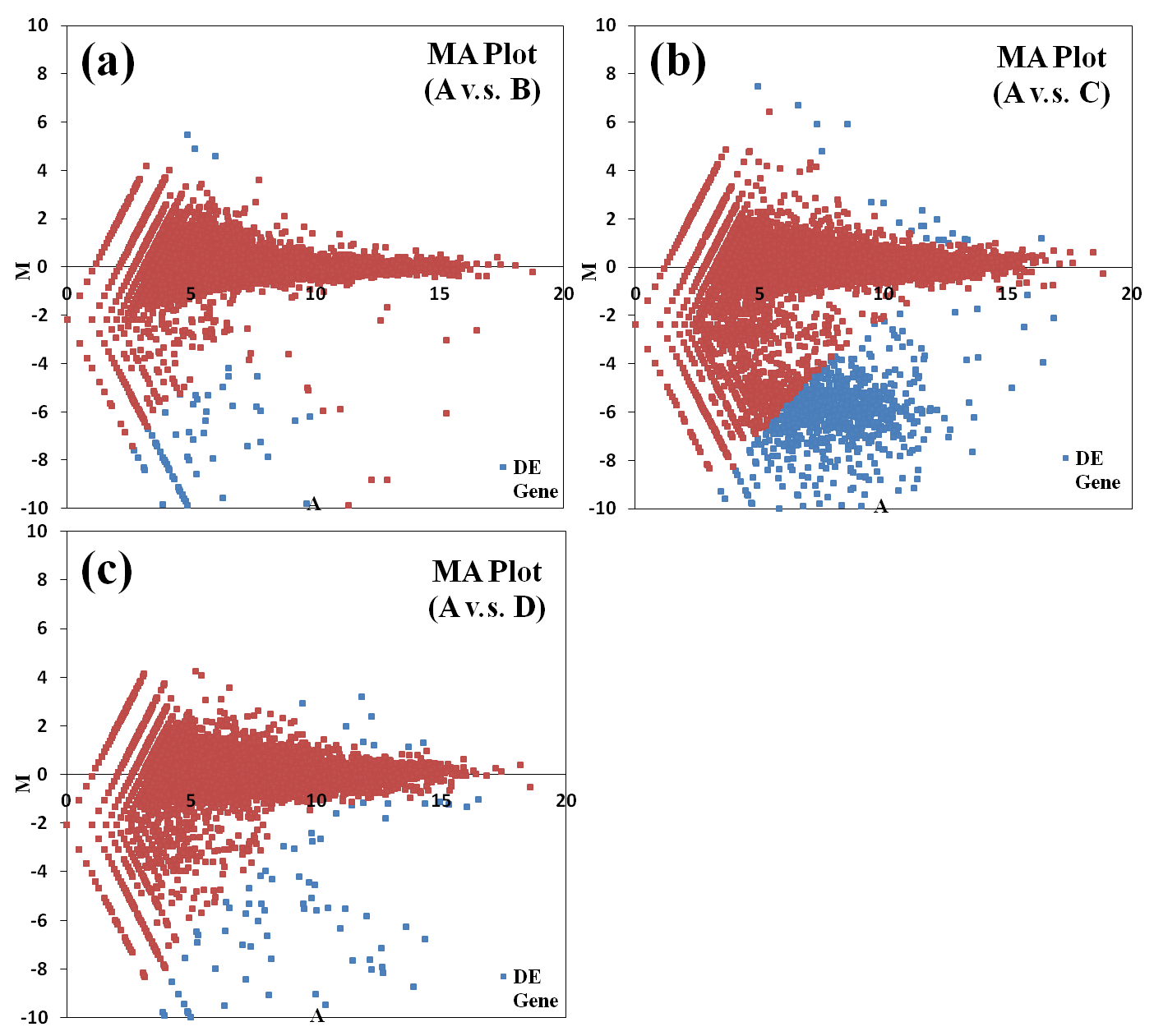


**Figure S13.** The “MA plot” pictures with log_2_(fold change) plotted versus baseMean foldchange of differentially expressed genes following exposure of *Scenedesmus* *obliquus* to silver. The letters of A, B, C and D represented the control, the 135μg L^-1^ r-AgNCs treatment, the 135μg L^-1^ r-AgNCs (contained 0.5 mM of L-cysteine) treatment and the 10μg L^-1^ silver ions treatment, respectively. Blue spots and red spots represented differentially expressed genes (︳old change︱>2 and *p*-value < 0.05) and no difference in genes expression, respectively.


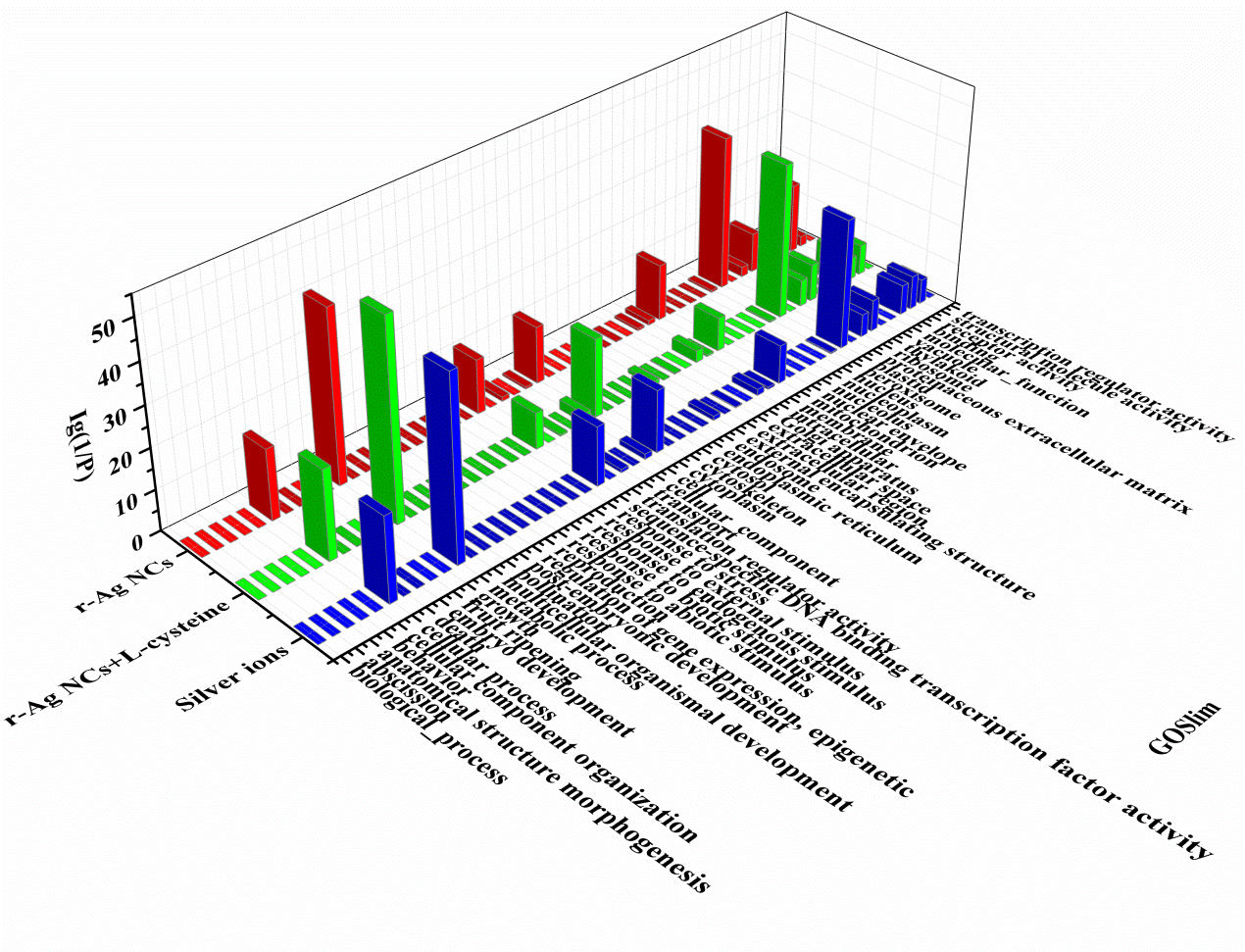


**Figure S14.** GO enrichment analysis of differentially expressed genes after exposure of *Scenedesmus* *obliquus* to silver for 96h. The enrichment analysis of differentially expressed genes was performed between each of silver treatment and the control (adjusted P values < 0.05) using all the unigenes as a background.

**Table S4.** List of differentially expressed genes of *Scenedesmus* *obliquus* after exposure of *Scenedesmus* *obliquus* to silver for 96h. Shades of red, light blue indicate down-regulation of target genes of each silver treatment compared with the control and no differential expression of genes (Unigenes) , respectively.

| **The light reaction of photosynthesis of *Scenedesmus* *obliquus*** | | | | | | |
| --- | --- | --- | --- | --- | --- | --- |
| **Photosystem II** | | | | | | |
| **Gene ID** | | **Gene Symbol** | **r-Ag NCs** | **r-Ag NCs + L-cysteine** | **Ag ions** | **Gene Description** |
| c112548_g1_i1 | | PsbA |  |  |  | Photosystem II P680 reaction center D1 protein |
| c40463_g1_i1 | | PsbC |  |  |  | Photosystem II CP43 chlorophyll apoprotein |
| c41384_g1_i1 | | PsbB |  |  |  | Photosystem II CP47 chlorophyll apoprotein |
| c45848_g1_i1 | | PsbO |  |  |  | Photosystem II oxygen-evolving enhancer protein 1 |
| **Photosystem I** | | | | | | |
| c68843_g1_i1 | | PsaA |  |  |  | Photosystem I P700 chlorophyll a apoprotein A1 |
| c30606_g1_i1 | | PsaD |  |  |  | Photosystem I subunit II |
| c23970_g1_i1 | | PsaE |  |  |  | Photosystem I subunit IV |
| c86827_g1_i1 | | PsaG |  |  |  | Photosystem I subunit V |
| c30092_g1_i2 | | PsaH |  |  |  | Photosystem I subunit VI |
| c18868_g1_i1 | | PsaL |  |  |  | Photosystem I subunit XI |
| c18510_g1_i1 | | PsaO |  |  |  | Photosystem I subunit PsaO |
| **Cytochrome b6/f complex** | | | | | | |
| c82867_g1_i1 | | PetA |  |  |  | Apocytochrome f |
| c72622_g1_i1 | | PetC |  |  |  | Cytochrome b6-f complex iron-sulfur subunit |
| **Photosynthethic electron transport** | | | | | | |
| c27650_g1_i1 | | PetE |  |  |  | Plastocyanin |
| c111825_g1_i1 | | PetF |  |  |  | Ferredoxin |
| c71270_g1_i1 | | PetH |  |  |  | Ferredoxin--NADP^+^ reductase |
| **F-type ATPase** | | | | | | |
| c21470_g1_i1 | | beta |  |  |  | F-type H+-transporting ATPase subunit beta |
| c40995_g21_i1 | | alpha |  |  |  | F-type H+-transporting ATPase subunit alpha |
| c111454_g1_i1 | | gamma |  |  |  | F-type H+-transporting ATPase subunit gamma |
| c6176_g1_i1 | | c |  |  |  | F-type H+-transporting ATPase subunit c |
| **The photosynthetic carbon reduction cycle of *Scenedesmus* *obliquus*** | | | | | | |
|  | **Gene ID** | **Gene Symbol** | **r-Ag NCs** | **r-Ag NCs + L-cysteine** | **Ag ions** | **Gene Description** |
| 4.1.1.39 | c86000_g1_i1 | rbcL |  |  |  | Ribulose-bisphosphate carboxylase large chain |
| 4.1.1.39 | c35349_g1_i1 | rbcs |  |  |  | Ribulose-bisphosphate carboxylase small chain |
| 2.7.23 | c18625_g1_i1 | PGK |  |  |  | Phosphoglycerate kinase |
| 1.2.1.12 | c31884_g1_i1 | GAPDH |  |  |  | Glyceraldehyde 3-phosphate dehydrogenase |
| 1.2.1.13 | c15020_g1_i1 | GAPA |  |  |  | Glyceraldehyde-3-phosphate dehydrogenase |
| 4.1.2.13 | c38135_g1_i1 | ALDO |  |  |  | Fructose-bisphosphate aldolase, class I |
| 5.3.1.1 | c22998_g1_i1 | TPI |  |  |  | Triosephosphate isomerase |
| 2.2.1.1 | c32559_g1_i1 | tktA |  |  |  | Transketolase |
| 4.1.2.13 | c38135_g2_i1 | ALDO |  |  |  | Fructose-bisphosphate aldolase, class I |
| 2.2.1.1 | c32559_g1_i1 | tktB |  |  |  | Transketolase |
| 5.1.3.1 | c114213_g1_i1 | rpe |  |  |  | Ribulose-phosphate 3-epimerase |
| 2.7.1.19 | c5346_g1_i1 | PRK |  |  |  | Phosphoribulokinase |

**Table S5.** Primer sequences for the qRT-PCR analyses.

| **Name** | **Sequences (5’ -> 3’)** | |
| --- | --- | --- |
| α-tubulin-F | Forward | GCTGTCTGATGTACCGTGGTGA |
|  | Reverse | CAGTGGGTGGCTGGTAGTTGAT |
| psbA | Forward | CGCATACCCAGACGGAAACT |
|  | Reverse | ATGGCTATACAACGGCGGTC |
| psbO | Forward | TCAGCACTAGCGGGTAAAGC |
|  | Reverse | CCACGGATTTTGAAGGCGAC |
| petF | Forward | CGCCTATCCCACCTCTGACT |
|  | Reverse | CGGGAGTTTTCTGACGATCTGG |
| ATPF0C | Forward | GGAGTCGGTCAAGGTACAGC |
|  | Reverse | GCGCTAATGCTACAACCAGG |
| rbcS | Forward | CGGTAAGGTCAGGAAGGT |
|  | Reverse | CGCAAGGCTAACAACGAC |

**References**

1. Yuan, X. *et al.* Highly luminescent silver nanoclusters with tunable emissions: cyclic reduction–decomposition synthesis and antimicrobial properties. *NPG Asia Mater.* **5,** e39, doi:10.1038/am.2013.3(2013).

2. Yuan, X. *et al.* Glutathione-protected silver nanoclusters as cysteine-selective fluorometric and colorimetric probe. *Anal. Chem.* **85,** 1913-1919, doi:10.1021/ac3033678(2013).

3. Zhang, L. *et al.* Uptake and effect of highly fluorescent silver nanoclusters on *Scenedesmus obliquus*. *Chemosphere* **153,** 322-331, doi:10.1016/j.chemosphere.2016.03.076(2016).
